# Supplementary material for: Proton-gated anion transport governs macropinosome shrinkage
Source: Nat Cell Biol. 2022 May 19;24(6):885–95. doi: 10.1038/s41556-022-00912-0 (PMC9203271; doi:10.1038/s41556-022-00912-0)
Supplement: Supplementary file 1 — Supplementary Note (mathematical model description) and Supplementary Note figures. [file 41556_2022_912_MOESM1_ESM.pdf]

nature portfolio

https://doi.org/10.1038/s41556-022-00912-0

Supplementary information

Proton-gated anion transport governs macropinosome shrinkage

In the format provided by the authors and unedited

Mathematical model

Description

We constructed a reductionist mathematical model for macropinosome resolution to check the plausibility of our mechanistic interpretations and to guide ongoing experiments. Macropinosomes were modeled as spheres with a luminal salt solution initially corresponding to the extracellular medium, and an initial transmembrane voltage equal to that of the plasma membrane. Whereas luminal ion concentrations were allowed to change as a consequence of transmembrane ion fluxes and changes in vesicle volume, outside ion concentrations were kept constant at values that are typical for the cytoplasm. Assuming infinite water permeability of the membrane, vesicle volume was set to be proportional to the luminal amount of osmotically active particles (*e.g.* ions). For simplicity, we assume that the number of ion transporters per membrane area is constant over time, thereby neglecting selective insertion or removal of transport proteins during macropinosome maturation. Constant membrane density of transporters results in a reduction of overall transport rate proportional to the decrease of the surface of the shrinking spherical vesicle (in the cell, reduction of vesicle surface is achieved by vesicle budding). Loss of luminal ions by vesicle budding was neglected. Depending on the parameters used for ion concentrations and conductances, our reductionist model predicts resolution to a final steady-state with a high luminal concentration of non-permeable ions, which is probably far from reality. However, for time points in which the predicted volume is sufficiently far from steady-state, our model provides a valuable plausibility check and can explain experimental results in a semi-quantitative manner. Insertion and removal of transport proteins during vesicle maturation could be easily introduced by multiplying the fluxes corresponding to the respective transporters with appropriate time-dependent factors, but given the dearth of relevant experimental data, our model neglects such processes. More realistic models require detailed knowledge of scission/fusion processes that affect the time-dependent abundance of relevant ion transporters, some of which may still be unknown, and detailed information on their biophysical properties.

Our model describes vesicles as spheres with an initial volume V_0 of

(1) $V_0 = 4/3 * \pi * r_0^3$

with r_0 being the initial radius,

and a surface area A_0 of

(2) $A_0 = 4 * \pi * r_0^2 = (36 * \pi)^{1/3} * V_0^{2/3}$

Assuming infinite water permeability, vesicle volume changes over time according to the number N_{osm} of luminal osmotically active particles:

(3) $V(t) = V_0 * N_{osm}(t) / N_{osm}(t=0)$

resulting in changes of the surface area

(4) $A(t) = (36 * \pi)^{1/3} * V(t)^{2/3}$

The vesicle has an electric capacitance C(t) given by:

(5) $C(t) = A(t) * c_{spec}$,

with c_{spec} being the specific capacity per membrane area.

We consider five ionic species: Cl^-, H^+, Na^+, K^+, and X, in which X (which may be any other ion, including buffers) is initially calculated from the given concentrations of the other species to yield the desired initial membrane voltage U (defined as potential difference to cytoplasm) according to:

(6) $U(t) = Q_{lum}(t) / C(t)$,

with Q_{lum} being the sum of the electrical charges in the lumen of the vesicle. The initial value of X is calculated as:

(7) $X_{lum}(t=0) = U(t=0) * C(t=0)/F – (Na^+_{lum}(t=0) + K^+_{lum}(t=0) + H^+_{tot\ lum}(t=0) – Cl^-_{lum}(t=0))$,

with F being Faraday's constant. The luminal *amounts* (moles) Y_{lum} refer to luminal *concentrations* $[Y]_{lum}$ by:

(8) $[X]_{lum}(t) = X_{lum}(t) / V(t)$, $[Na^+]_{lum}(t) = Na^+_{lum}(t)/V(t)$, $[Cl^-]_{lum}(t) = Cl^-_{lum}(t)/V(t)$, etc.

Special attention must be given to H^+ since the majority of protons binds to buffers, with free H^+ concentration $[H^+_{free}]$ being several orders of magnitude smaller than the total concentration $[H^+_{tot}]$. The relationship between both concentrations is given by a constant reflecting an approximate buffer capacity β^*:

(9) $[H^+_{free}](t) = [H^+_{tot}](t) * 1/\beta^*(t)$.

Since vesicle shrinkage increases $[H^+_{tot}]_{lum}$, the lumen acidifies also without transmembrane H^+ flux if the buffer capacity remains constant. However, the bulk of $[H^+_{tot}]_{lum}$ is bound to buffers, the concentration of which also increases during shrinkage. Therefore, we put:

(10) $\beta^*(t) = \beta^*(t=0) * (V_0/V(t))$,

which eliminates purely shrinkage-driven acidification.

pH is calculated as usual:

(11) $pH = - \log_{10}([H^+_{free}])$.

The amount of free H^+ (H^+_{free}) is negligible compared to that of other ions and thus must not be considered for the osmotic shrinkage of the vesicle. The number of osmotic particles in the lumen ($N_{osm\ lum}$), which determines the size of the vesicle according to eq. (3), is:

(12) $N_{osm\ lum}(t) = Na^+_{lum}(t) + K^+_{lum}(t) + Cl^-_{lum}(t) + X_{lum}(t)$.

Note that the transport of H^+ into the vesicle in stoichiometric exchange for a different luminal ion may lead to vesicle shrinkage because the osmotic effect of H^+ is abolished by binding to buffer.

We model vesicles as having various combinations of Cl^- channels (ASOR), Na^+ channels (TPCs), $2Cl^-/H^+$-exchangers (CLCs such as ClC-5) and a proton pump (H^+-ATPase). As previously[1], we assume for simplicity that transport rates are proportional to the driving force provided by the respective ion concentration differences and transmembrane voltage (and additionally ATP hydrolysis in the case of the proton pump). To model ASOR and CLCs we additionally multiply by equations that semi-quantitatively describe their steep voltage- and pH-dependencies.

We write for the respective ion fluxes (positive when directed into the lumen):

Flux through Na^+ channels (TPCs):

(13) $J_{Na} = (-U + RT/F * \ln([Na^+]_{cyt}/[Na^+]_{lum})) * g_{TPC} * A$

Flux through voltage- and pH-dependent Cl^- channels (ASOR):

(14) $J_{Cl\ ASOR} = (U + RT/F * \ln([Cl^-]_{cyt}/[Cl^-]_{lum})) * g_{ASOR} * f_{ASOR}(U) * f_{ASOR}(pH) * A$

Cl^- flux through voltage- and pH-dependent $2Cl^-/H^+$-exchangers (ClC-5):

(15) $J_{Cl\ CLC} = 2 * (U + RT/3F * \ln(([Cl^-]_{cyt}/[Cl^-]_{lum})^2 * ([H^+_{free}]_{lum}/[H^+_{free}]_{cyt}))) * g_{CLC} * f_{CLC}(U) * f_{CLC}(pH) * A$

H^+ flux through voltage- and pH-dependent $2Cl^-/H^+$-exchangers (ClC-5):

(16) $J_{H\ CLC} = - (U + RT/3F * \ln(([Cl^-]_{cyt}/[Cl^-]_{lum})^2 * ([H^+_{free}]_{lum}/[H^+_{free}]_{cyt}))) * g_{CLC} * f_{CLC}(U) * f_{CLC}(pH) * A$

For the H^+-ATPase, we wrote simplified as in[1]:

(17) $J_{H\ ATP} = (U_{ATP} - U - RT/F * \ln([H^+_{free}]_{lum}/[H^+_{free}]_{cyt})) * g_{ATP} * A$

and for the H^+-conductance ('proton leak'):

(18) $J_{H\ leak} = (-U - RT/F * \ln([H^+_{free}]_{lum}/[H^+_{free}]_{cyt})) * g_{H\ leak} * A$

g_{TPC}, g_{ASOR}, g_{CLC} , g_{ATP} and $g_{H\ leak}$ are scaling factors (dimension mol/(V * sec * m^2)), U_{ATP} the electrochemical potential reached by ATP hydrolysis (270 mV) [2], and R,T, and F the gas constant, absolute temperature, and Faraday's constant respectively. A is the surface area of the vesicle which changes over time with shrinkage as given by (3) and (4).

Functions f describe the voltage- and pH-dependence of ASOR and CLC. They can assume values between 0 and 1 and have the general form:

(19) $f(pH) = 1 / (1 + e^{(k1*(pH-pH1/2)})$ and

(20) $f(U) = 1 / (1 + e^{(k2*(U-U1/2)})$

with $pH_{1/2}$ and $U_{1/2}$ being values of pH and voltage, respectively, where half maximal activation is achieved.

For simplicity, macropinosomal Na^+ channels, likely embodied by both TPC1 and TPC2[3], were modeled as being voltage- and pH-independent. Whereas TPC1 currents are strongly voltage-dependent[4,5], this is not the case for TPC2, but rectification and other properties of either channel strongly depend on the endogenous or artificial agonist used for their activation[4,6,7]. Whereas luminal alkalinization increases TPC1-mediated Na^+ currents[5], it decreases TPC2-mediated Ca^{2+} currents[7]. Given these uncertainties, we opted for modeling Na^+ channels as unregulated 'leak' currents. Likewise, we modeled the H^+-conductance as an unregulated 'leak'.

Luminal amounts of ions were calculated by numerical integration (using Python and stiff Euler integration) of the differential equations:

(21) $dCl^-_{lum}/dt = J_{Cl\ ASOR} + J_{Cl\ CLC}$

(22) $dH^+_{tot\ lum}/dt = J_{H\ CLC} + J_{H\ ATP} + J_{H\ leak}$

(23) $dNa^+_{lum}/dt = J_{Na}$

At each integration step, V(t), A(t), U(t) and pH(t) were calculated according to the above equations and ion concentrations were obtained by dividing ion amounts by the actual volume V(t). This was also done for the non-transported species such as X, increasing luminal concentrations with shrinkage.

Choice of parameters

For the calculations shown in Supplementary Note Figs. 1 – 4, the following parameters were used:

c_{spec} = 0.01 Farad/m^2, the typical capacitance of biological membranes [8].

$1/\beta(t=0) = 5 * 10^{-4}$, corresponding to an approximate buffer capacity of 2.5 mM H^+_{tot} /pH unit, which is low compared to that for cytosol (usually thought to be ≈ 30 mM H^+_{tot}/pH unit), but has the right order of magnitude for our experiments in which uptake buffer contained 20 mM HEPES which displays reduced buffering at pH < 6.8.

r_0 = 1.3 µm; U(t=0) = +40 mV (corresponding to -40 mV plasma membrane voltage in the usual convention).

$[Cl^-]_{cyt}$ = 20 mM; $[Na^+]_{cyt}$ = 10 mM; $[K^+]_{cyt}$ = 140 mM; pH_{cyt} = 7.2

$[Cl^-]_{lum}$ = 159 mM; $[Na^+]_{lum}$ = 150 mM; $[K^+]_{lum}$ = 5 mM; pH_{lum} = 7.4

Unless otherwise indicated, we used the following parameters to approximate the steep outward rectification of both ClC-5 and ASOR, and the opposite pH-dependencies of both transport proteins:

$f_{CLC}(U) = 1/(1 + e^{80 * (U(t) + 0.04)})$, and similarly $f_{ASOR}(U) = 1/(1 + e^{80 * (U(t) + 0.04)})$, resulting in a steep voltage dependence with half-maximal activation at luminal -40 mV.

$f_{ASOR}(pH) = 1/(1 + e^{3* (pHlum - 5.4)})$ and $f_{CLC}(pH) = 1/(1 + e^{1.5* (5.5 - pHlum)})$.

These function give the pH- and voltage-dependencies shown in Supplementary Note Fig. 2 that roughly approximate experimental data for ASOR[9-11] and ClC-5[12,13].

To estimate the order of magnitude for ion flux through ASOR per unit, we considered published values for ASOR plasma membrane currents in different cell lines [10,11,14], and took a value of I/C = 100 pA/pF at + 100 mV, *i.e.* $I/(U*C) = 10^3$ S/F as estimate for maximally activated ASOR conductance per membrane capacitance. Since the specific membrane capacitance is c_{spec} = 0.01 F/m^2, this translates to 10 S/m^2. Converting this value from electrical conductance to substance flow, we divide by Faraday's constant F = 96 485 to obtain $g_{ASOR} \sim 1* 10^{-4}$ moles/(sec * m^2 * V). This rough estimate rests on the assumptions that channel density per surface area does not differ between the plasma membrane and macropinosomes and that channel properties are not changed by differences in membrane composition. The corresponding values g_{TPC}, g_{CLC}, g_{ATPase} and $g_{H\ leak}$ were arbitrarily adjusted to yield macroscopic fluxes of the same order of magnitude as J_{ASOR}, while allowing ASOR to yield ≈ 5-fold more maximal currents than CLCs to account for the generally higher

conductance of channels vs. transporters. Given the strong voltage- and pH dependencies of ASOR and ClC-5, this approach required smaller values for g_{TPC} and g_{ATPase}.
Thus, for the calculations of the 'complete' model in Supplementary Note Fig. 3 we took the following scaling factors: $g_{ASOR} = 8*10^{-5}$, $g_{TPC} = 2*10^{-6}$, $g_{CLC} = 1*10^{-7}$, $g_{ATP} = 8*10^{-9}$, and $g_{H\ leak} = 1.6*10^{-8}$.

Model calculations

To obtain basic insights into effects of transport processes, we first examined oversimplified models in which none of the transporters displays regulation by pH or voltage, with transmembrane ion fluxes being proportional to the driving force given by the Nernst potential (Supplementary Note Fig. 1). We used the same scaling factors g as for subsequent calculations in which we introduced voltage- and pH-dependencies for ASOR and CLCs, and set the respective factors f describing these dependencies as being constantly 0.5. The oversimplified models (Supplementary Note Fig. 1) reveal the fundamental effects of transporters and channels that lack explicit voltage- and pH dependencies and to correlate the predicted changes in pH, voltage, and volume with ion fluxes through specific transporters.

Supplementary Note Fig. 2 depicts the voltage- and pH-dependencies of ASOR and CLC calculated from (19) and (20) with parameters that approximate published experimental data.

Simulations in Supplementary Note Fig. 3 explore the model in Fig. 7a that includes ASOR, TPC, CLC, H^+-leak and H^+-ATPase, with CLCs and ASOR displaying the pH- and voltage-dependencies shown in Supplementary Note Fig. 2. This reductionist model, in which expression levels of transporters stay constant over time (and thus ignores insertion and retrieval of transporters from the MP membrane) suggests that CLCs have the greatest impact on pH, U and resolution during the first few minutes after MP formation. Unfortunately, owed to the necessity of exposing the cells to M-CSF and fluorescent TMR-dextran for a few minutes and having to thoroughly wash away the dye afterwards, we cannot measure in this time window in our experiments.

Calculations in Supplementary Note Fig. 4 explore negative feedback loops impinging on ASOR activity, in the presence of pH- and V-dependent ASOR and CLC, and of TPCs and V-type-ATPases. Supplementary Note Fig. 4a explores the effect of increasing the expression of

‘WT’ ASOR 5-fold, and of a 5-fold increase of an ASOR mutant showing a wider and alkaline-shifted pH dependence (see Supplementary Note Fig. 2). Whereas overexpression of ‘WT’ ASOR has a moderate effect on pH, U, and volume V, the mutant markedly accelerates resolution (as observed in the experiments shown in Fig. 7c), makes the lumen more alkaline and shift the luminal potential to more positive values. Both parameters feed back negatively on ASOR currents, rendering resolution remarkably resilient towards ASOR expression levels.

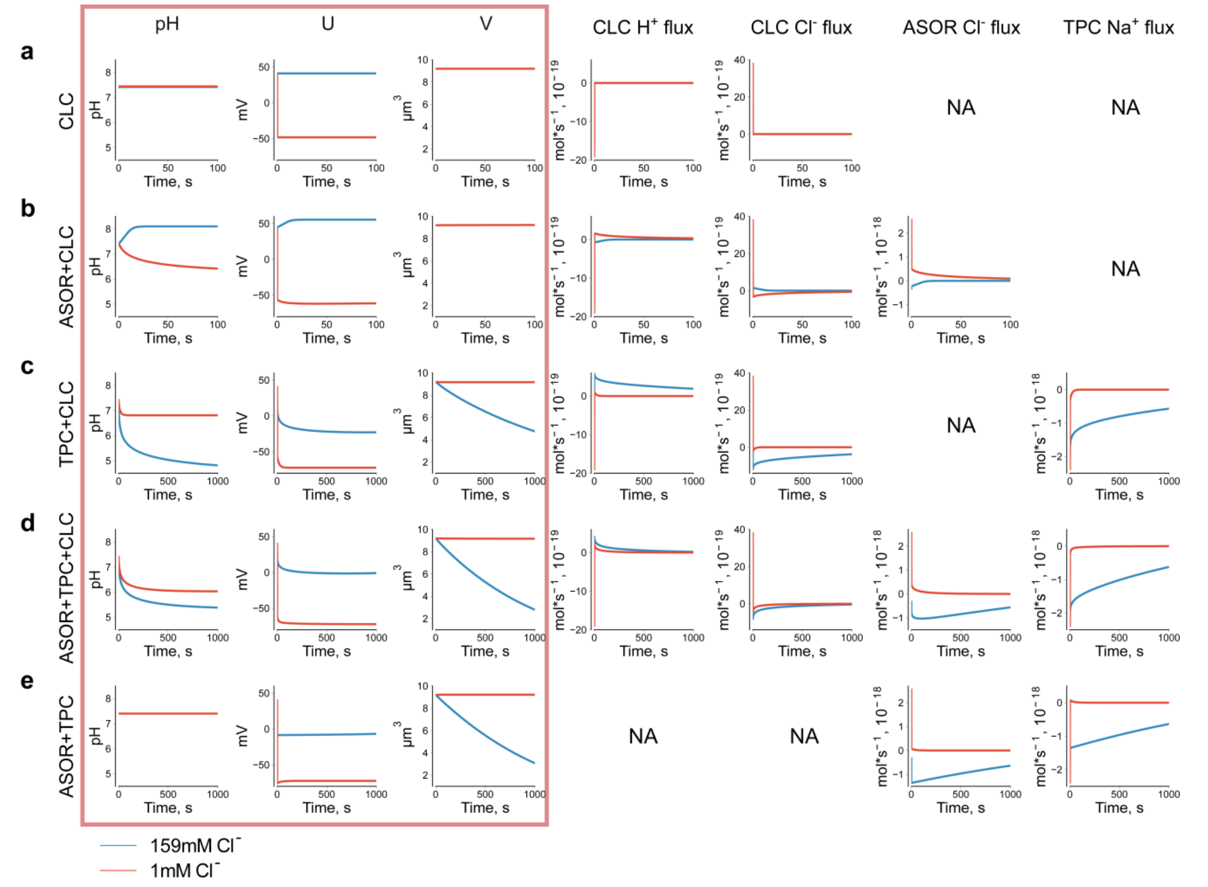

Supplementary Note Figure 1. Calculations for simplified vesicle models containing *voltage- and pH-independent* 2Cl$^-$/H$^+$-exchangers ('CLC'), Cl$^-$ channels ('ASOR') and Na$^+$ channels ('TPC').

Calculations with initial high (159 mM) or low (1 mM) luminal Cl$^-$ concentrations ($[Cl^-]_{lum}$). Results of model calculations are shown for luminal pH, membrane potential U (referred to cytoplasm), vesicle volume V, as well as ion fluxes (when applicable) through CLCs, ASOR, and TPC over a time span of 100 (a,b) or 1000 s (c-e). **(a)** Vesicles containing only a 'CLC' 2Cl$^-$/H$^+$-exchanger quickly reach an equilibrium with inside-positive potential with high luminal Cl$^-$, and a luminal negative potential with low chloride. No significant change of luminal pH because H$^+$ is much more efficiently buffered than electrical charge (by membrane capacitance). Virtually no change in vesicle volume. **(b)** Parallel operation of a 'CLC' and a Cl$^-$ conductance 'ASOR'. Voltage changes with Cl$^-$ gradients are larger than in (a) because Cl$^-$ gradients produce a 3/2-fold larger change in electrochemical potential with a Cl$^-$ channel than with a 2Cl$^-$/H$^+$-exchanger, as evident from respective Nernst equations. At these voltages, the CLC is initially far from equilibrium, leading to CLC-mediated H$^+$- transport that lead to luminal alkalinization and acidification with high and low luminal $[Cl^-]_{lum}$, respectively, until an equilibrium is reached. The change is pH appears at first counterintuitive, as one would think that an inside-out Cl$^-$ gradient would increase luminal [H$^+$] by exchanging external H$^+$ for luminal Cl$^{.-}$ through CLC 2Cl$^-$/H$^+$-exchange - however, and alkalinization is predicted. This effect is explained by the effect of the 'ASOR' Cl$^-$ conductance on U, which changes the transport direction of the CLC. Virtually no change in volume as Cl$^-$ fluxes are not

electrically neutralized by cation currents. **(c)** Parallel operation of a $2Cl^-/H^+$-exchanger and a Na^+ channel. TPC-mediated Na^+ efflux renders the lumen more negative, the specific voltage depending on the ratio of CLC over TPC conductance. The lumen-negative potential drives CLC-mediated Cl^- efflux coupled to H^+ influx, which is of course larger with high luminal Cl^-. Note that with high Cl^- there is strong luminal *acidification*, contrasting with the moderate *alkalinization* predicted with CLC + ASOR under the same ionic conditions (b). This difference is largely due to the opposing effects of Na^+ and Cl^- channels on U and explains, in principle, the opposite pH changes with low luminal Cl^- with WT and *Tmem206*$^{-/-}$ macropinosomes (Fig. 5a, e). **(d)** Parallel operation of ASOR + TPC + CLC. Luminal acidification is again stronger with higher than with lower $[Cl^-]_{lum}$, mainly because of the changes in U (that depend on the relative conductances of all three transporters). The uncoupled Cl^- transport pathway provided by the Cl^- channel leads to more vesicle shrinkage. **(e)** Parallel operation of Cl^- and Na^+ channels leads to vesicle shrinkage with high, but not low $[Cl^-]_{lum}$. No change in pH as either channel transports H^+.

Parameters for calculation: Total time of simulation is 100 s (a-b) or 1000 s (c-e) with 0.001 s step. General parameters as in 'Choice of parameters'. The following values g for the 'strength' of transporters (in $mol*s^{-1}*V^{-1}*m^{-2}$, see equations (13)-(16)) were used: (a) $g_{CLC} = 4*10^{-6}$. (b) $g_{ASOR} = 4*10^{-6}$, $g_{CLC} = 4*10^{-6}$. (c) $g_{TPC} = 1*10^{-6}$, $g_{CLC} = 4*10^{-6}$. (d) $g_{ASOR} = 4*10^{-6}$, $g_{TPC} = 1*10^{-6}$, $g_{CLC} = 4*10^{-6}$. (e) $g_{ASOR} = 4*10^{-6}$, $g_{TPC} = 1*10^{-6}$. For the other transporters, the respective value of g was set to 0.

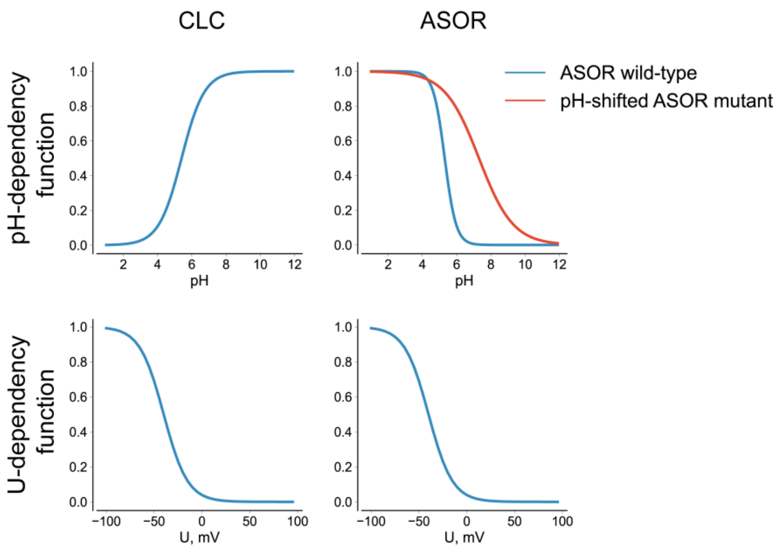

Supplementary Note Figure 2. Effects of voltage- and pH-dependencies of ASOR and CLCs. Graphical depiction of pH- and voltage-dependencies of CLCs (left) and ASOR (right) used in our model calculations to approximate experimental data for vesicular CLCs[12,15-17] and ASOR[9,11,18,19]. Equations are of the form $f(pH) = 1 / (1 + e^{(k1*(pH-pH1/2)})$ and $f(U) = 1 / (1 + e^{(k2*(U-U1/2)})$, with k_1=3, $pH_{1/2}$=5.4 for wild-type ASOR, k_1= -1.5, $pH_{1/2}$=5.5 for ClC; k_2=80, $U_{1/2}$= -40 mV. We also mimicked a pH-shifted mutant TMEM206(R87C) [11] (red line) for which we chose k_1=1 and $pH_{1/2}$=7.4.

Supplementary Note Figure 3. Role of individual ion transporters explored in vesicle model considering voltage- and pH dependencies of ASOR and CLC.

A vesicle was modeled to contain 'ASOR' Cl^- channels and 'CLC' $2Cl^-/H^+$-exchangers, both described to be pH- and voltage-regulated, as well as an unregulated Na^+-conductance ('TPC'), an unregulated H^+-conductance and an H^+-ATPase, using simplified equations in which fluxes are proportional to the electrochemical driving force and parameters as specified in 'model description'. Calculations that explored the impact of luminal Cl^--concentrations (159 *vs* 9 mM as on our experiments) were performed for:

(a) the complete model vesicle (see Fig. 7a).

(b - d) vesicles lacking the Cl^- conductance ('ASOR KO'), also together with 'deletions' of the H^+-ATPase (**c**), or of both H^+-ATPase and H^+-leak (**d**).

(e - g) vesicles *lacking* only one of the three H^+-transporters, *i.e.* CLC (**e**), H^+-ATPase (**f**), or H^+-leak (**g**).

(h – j) vesicles *expressing* only one of the H^+-transporters, *i.e.* CLC (**h**), H^+-ATPase (**i**) or H^+-leak (**j**).

(k) vesicles *expressing* none of the H^+-transporters.

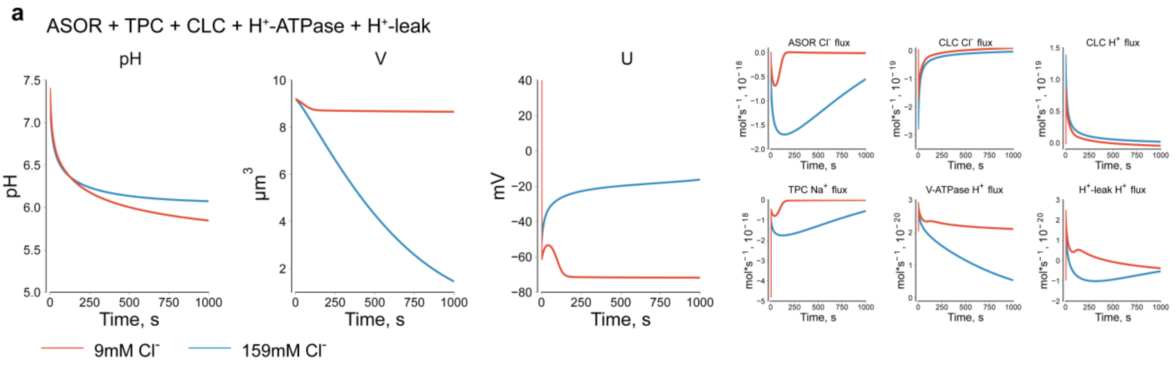

(a) Simulation for complete model vesicle (Fig. 7a) with high or low $[Cl^-]_{lum}$ (blue line and red lines, respectively). Left three panels show luminal pH, volume V, and voltage U, with smaller right panels displaying Na^+- , Cl^-- and H^+-fluxes through respective transporters as function of time. Vesicles are acidified with both high and low luminal Cl^-. Vesicles are more acidic under low than under high Cl^- conditions, as observed experimentally (Fig. 5a). This relative acidification is caused by the large difference in luminal potential caused by ASOR-mediated Cl^- currents that render the lumen ≈50 mV more negative with low compared to high $[Cl^-]_{lum}$, This difference in U increases the driving force for electrogenic H^+ uptake with low *vs.* high $[Cl^-]_{lum}$. Vesicle models lacking various H^+-transporters suggest that the acidification by Cl^- removal occurs in the presence of either an H^+-ATPase or an H^+-leak (see panels e, f, g, i, j below), but not when CLCs are the only H^+ transporters (h). Note that net ion flow through CLC $2Cl^-/H^+$-exchangers is most prominent during the first minutes. As expected and found experimentally (Fig. 1c, d), low $[Cl^-]_{lum}$ almost completely abolishes resolution that normally proceeds at nearly unchanged rate for a prolonged time.

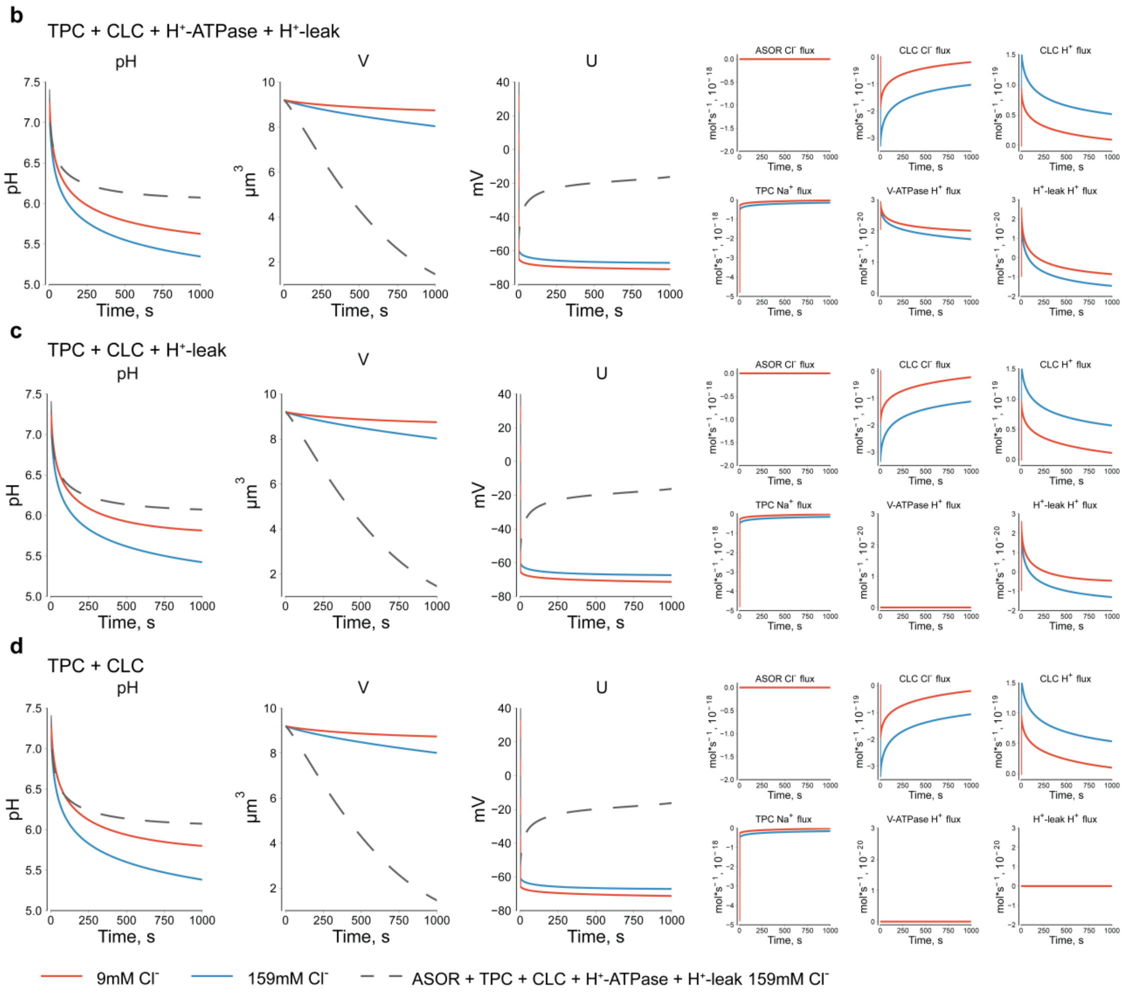

(b) Effect of deleting the 'ASOR' Cl^- channel. Luminal voltage U is now negative irrespective of $[Cl^-]_{lum}$. It is largely determined by 'TPCs' and the inside-out Na^+-gradient. ASOR KO leads to more acidic pH_{lum} is more acidic than in the 'WT' under high $[Cl^-]_{lum}$,, which is shown for comparison (taken from(a)), as observed experimentally (Fig. 5a). In contrast to 'WT' (a), and as observed (Fig. 5e), lowering $[Cl^-]_{lum}$ leads to relative alkalinization. Both effects can be attributed to CLC $2Cl^-/H^+$-exchange, which accumulates H^+ in the lumen in exchange for luminal Cl^- in the presence of high $[Cl^-]_{lum}$, but not in its absence. This change in pH_{lum} occurs also in the absence of an H^+-ATPase (c) and an H^+-conductance ('leak') (d). The experiment of Fig. 5e thus strongly indicates the presences of a CLC $2Cl^-/H^+$-exchanger, likely ClC-5, on MPs. Note that the combination TPC/CLC can support vesicle shrinkage in the absence of ASOR, although at a much reduced rate. Indeed, even overexpression of ClC-5, which prominently localized to MPs, could not compensate for the loss of TMEM206 (Fig. 4j).

(c) Calculation for vesicle without 'ASOR' and H^+-ATPase as model for *Tmem206*$^{-/-}$ MPs in the presence of bafilomycin (Fig. 5d). Absence of proton pump activity leads to slight alkalinization compared to (b), and ΔpH_{lum} between high and low $[Cl^-]_{lum}$ is similar to (b).

(d) Vesicle lacking 'ASOR', H^+-ATPase and H^+-leak. The relative alkalinization by luminal Cl^- removal can only be attributed to CLC $2Cl^-/H^+$-exchange.

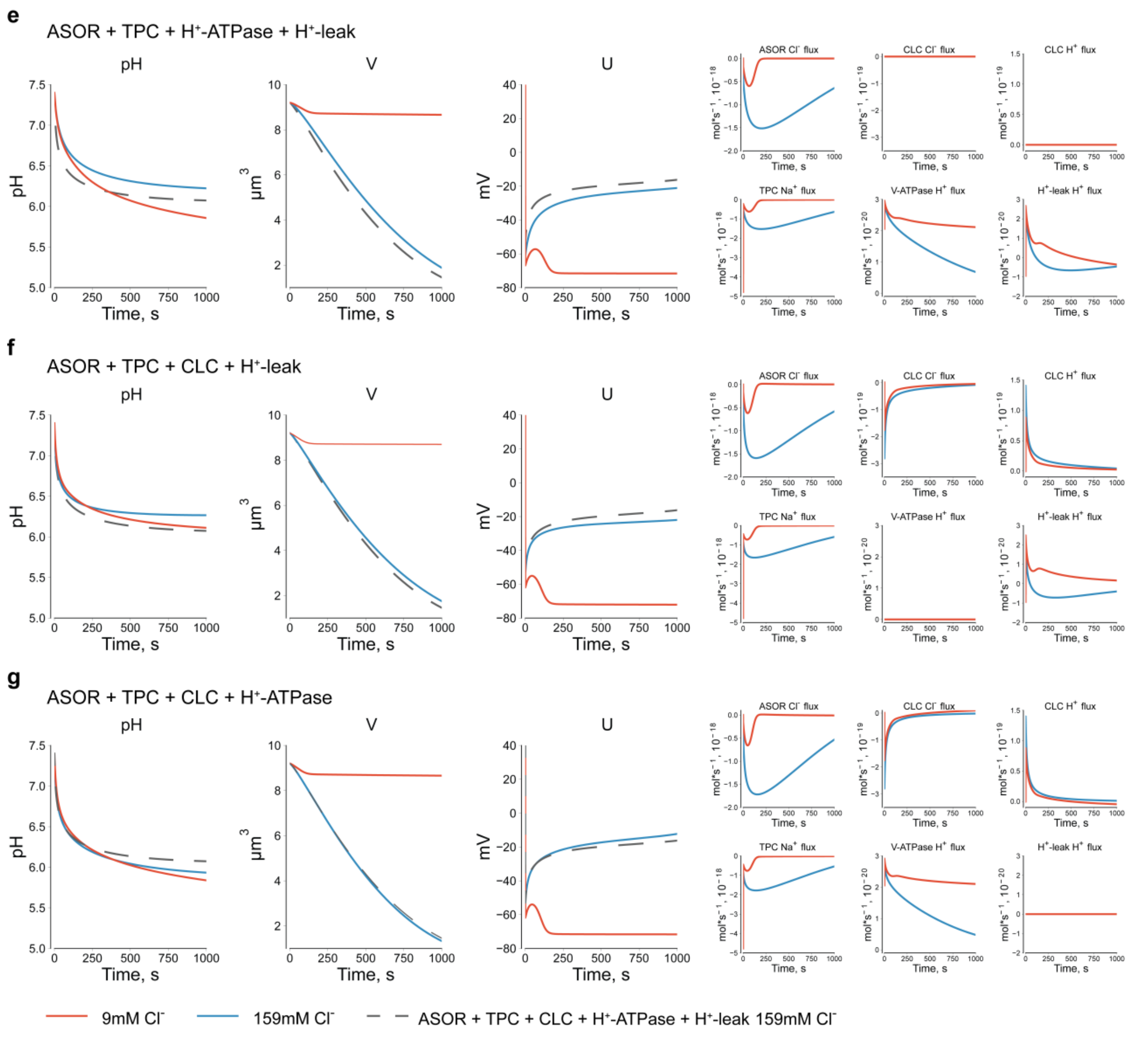

(e) Vesicle lacking CLC exchanger (blue lines) shows slightly delayed and reduced acidification and resolution compared to 'WT' (black dashed lines, from a). Low $[Cl^-]_{lum}$ (red line) leads to relative acidification, primarily owing to changes in luminal potential as in (a).

(f) Vesicle lacking H^+-ATPase (blue lines) shows reduced acidification and slightly reduced resolution compared to 'WT' (black dashed lines, from a). Low $[Cl^-]_{lum}$ (red line) again leads to relative acidification.

(g) Vesicle lacking H^+-leak (blue lines) shows enhanced acidification and slightly increased resolution compared to 'WT' (black dashed lines, from a). Low $[Cl^-]_{lum}$ (red line) again leads to relative acidification.

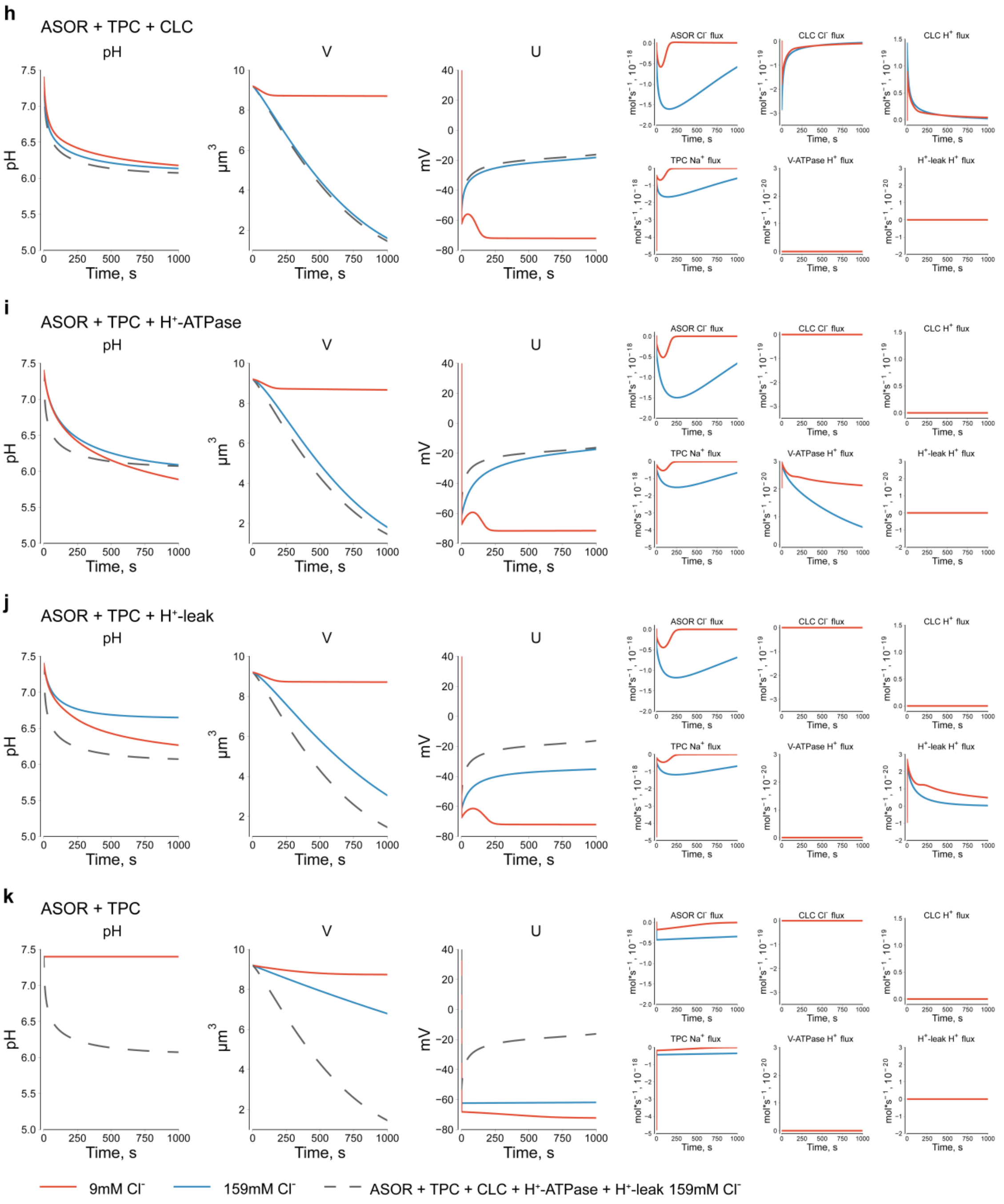

(h) Vesicle expressing CLC as only H^+-transporter (blue lines) show almost unchanged acidification and resolution compared to 'WT' (black dashed lines, from a). Importantly, low $[Cl^-]_{lum}$ (red line) does not lead to relative acidification, because with the CLC exchanger the outside-in Cl^- gradient counteracts the increased driving force for electrogenic H^+ entry provided by the ASOR- and TPC-generated lumen-negative potential.

(i) Vesicle expressing H^+-ATPase as only H^+-transporter (blue lines) shows delayed, but in the end enhanced acidification and slightly delayed, but in the end normal resolution compared to 'WT' (black dashed lines, from a). Low $[Cl^-]_{lum}$ (red line) leads to relative acidification owing to the more negative luminal potential

(j) Vesicle expressing an H^+-leak as only H^+-transporter (blue lines) shows markedly acidification and only moderately reduced resolution compared to 'WT' (black dotted lines, from a). Low $[Cl^-]_{lum}$ (red line) leads to relative acidification due to the negative luminal potential. This vesicle models *Clcn5*$^{-/-}$ MPs in the presence of bafilomycin (Fig. 5f, g)

(k) Vesicle lacking any acidifying transport process (blue lines) shows markedly reduced resolution compared to 'WT' (black dashed lines, from a) or compared to a model vesicle expressing a 'proton leak' (j). Even with the strong pH-dependence of ASOR, as modeled in Supplementary Note Fig. 2, ASOR currents are non-zero. In our experiments with NH_4Cl (Fig. 5b, c) the lumen most likely achieves more alkaline pH, leading to a virtual shutdown of ASOR and resolution.

Conclusion from calculations in Supplementary Note Fig. 3:

ASOR and TPC are essential for an efficient resolution of the model vesicle. While Cl^- transport through CLCs can, in principle, partially replace ASOR in this task, it is much less efficient, as experimentally confirmed by ClC-5 overexpression in *Tmem206*$^{-/-}$ BMDMs. Given the pH-dependence of acid-sensitive ASOR/TMEM206 channels, resolution depends on luminal acidification which can occur through several mechanisms. Even an H^+-conductance, which acts as 'proton leak' with sufficiently acidic lumen (generated *e.g.* by the H^+-ATPase), can acidify the lumen to a degree that yields a resolution rate that is only moderately smaller than in the 'WT' model. This renders resolution remarkably insensitive to the amount and nature of different acidifiers. This resilience is caused by negative feedback loops generated by the steep voltage- and pH-dependencies of ASOR and CLCs (see Supplementary Note Fig. 4).

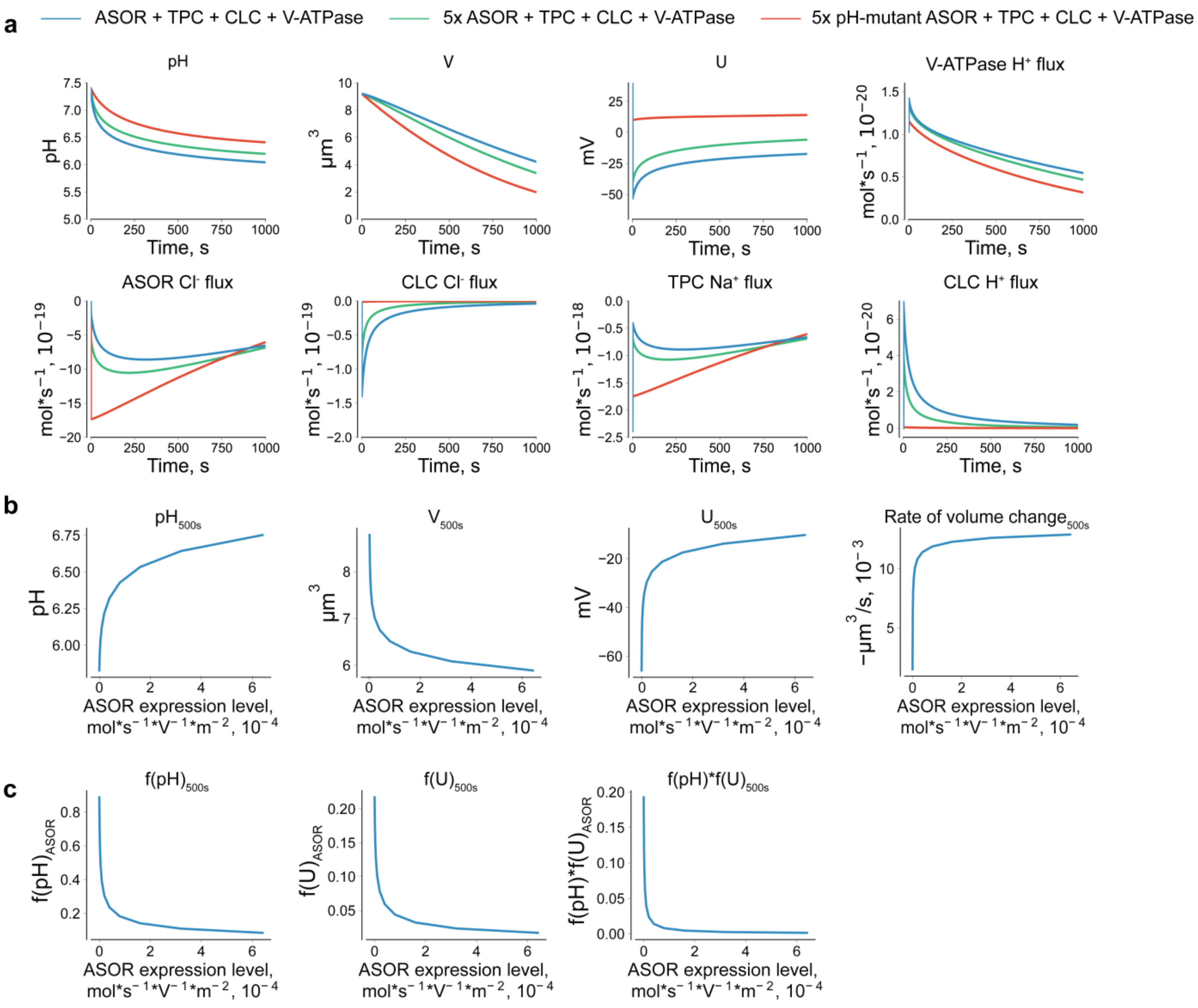

Supplementary Note Figure 4. Effect of ASOR expression levels and an ASOR mutant with changed pH-dependence.

(a) Calculations examining the effect of 5-fold increase of ASOR expression, either WT or pH-shifted mutant (Supplementary Note Fig. 2), in the presence of TPC, CLC, and V-type ATPase. Note moderate effect of WT ASOR overexpression, and a much stronger effect of the mutant which is accompanied by an alkaline shift in pH_{lum} and a shift to lumen-positive potentials. **(b)** Plot of pH $_{lum}$, voltage U $_{lum}$, volume V and shrinkage rate (dV/dt) at 500 s as function of ASOR expression levels. **(c)** Plot of $f(pH)_{ASOR}$, $f(U)_{ASOR}$ (eq. (19) and (20) in model description) and $f(pH)_{ASOR}$ * $f(U)_{ASOR}$ at t=500 s as measure of negative feed-back on ASOR activity. Note the contribution of both pH and U in suppression of ASOR currents with higher ASOR expression levels.

Parameters used for calculations: Total time of simulation, 1000s with 0.001s step, general parameters as in 'Choice of parameters'. (a) $g_{ASOR}(1x) = 4*10^{-5}$, $g_{ASOR}(5x) = 2*10^{-4}$, $g_{TPC} = 1*10^{-6}$, $g_{CLC} = 5*10^{-8}$, $g_{ATP} = 4*10^{-9}$ (b-c) 1x ASOR expression level $g_{ASOR} = 4*10^{-5}$, changed in 2-fold steps as indicated; $g_{TPC} = 1*10^{-6}$, $g_{CLC} = 5*10^{-8}$, $g_{ATP} = 0$.

Supplementary note references

1 Weinert, S. *et al.* Lysosomal pathology and osteopetrosis upon loss of H^+-driven lysosomal Cl^- accumulation. *Science* **328**, 1401-1403, doi:10.1126/science.1188072 (2010).

2 Rybak, S. L., Lanni, F. & Murphy, R. F. Theoretical considerations on the role of membrane potential in the regulation of endosomal pH. *Biophys J* **73**, 674-687, doi:10.1016/S0006-3495(97)78102-5 (1997).

3 Freeman, S. A. *et al.* Lipid-gated monovalent ion fluxes regulate endocytic traffic and support immune surveillance. *Science* **367**, 301-305, doi:10.1126/science.aaw9544 (2020).

4 Zhang, X. *et al.* Agonist-specific voltage-dependent gating of lysosomal two-pore Na^+ channels. *eLife* **8**, doi:10.7554/eLife.51423 (2019).

5 Cang, C., Bekele, B. & Ren, D. The voltage-gated sodium channel TPC1 confers endolysosomal excitability. *Nature chemical biology* **10**, 463-469, doi:10.1038/nchembio.1522 (2014).

6 Gerndt, S. *et al.* Agonist-mediated switching of ion selectivity in TPC2 differentially promotes lysosomal function. *eLife* **9**, doi:10.7554/eLife.54712 (2020).

7 Schieder, M., Rötzer, K., Brüggemann, A., Biel, M. & Wahl-Schott, C. A. Characterization of two-pore channel 2 (TPCN2)-mediated Ca^{2+} currents in isolated lysosomes. *J Biol Chem* **285**, 21219-21222, doi:10.1074/jbc.C110.143123 (2010).

8 Gentet, L. J., Stuart, G. J. & Clements, J. D. Direct measurement of specific membrane capacitance in neurons. *Biophys J* **79**, 314-320, doi:10.1016/S0006-3495(00)76293-X (2000).

9 Lambert, S. & Oberwinkler, J. Characterization of a proton-activated, outwardly rectifying anion channel. *The Journal of physiology* **567**, 191-213, doi:10.1113/jphysiol.2005.089888 (2005).

10 Wang, H. Y., Shimizu, T., Numata, T. & Okada, Y. Role of acid-sensitive outwardly rectifying anion channels in acidosis-induced cell death in human epithelial cells. *Pflügers Arch* **454**, 223-233, doi:10.1007/s00424-006-0193-z (2007).

11 Ullrich, F. *et al.* Identification of TMEM206 proteins as pore of PAORAC/ASOR acid-sensitive chloride channels. *eLife* **8**, doi:10.7554/eLife.49187 (2019).

12 Friedrich, T., Breiderhoff, T. & Jentsch, T. J. Mutational analysis demonstrates that ClC-4 and ClC-5 directly mediate plasma membrane currents. *J Biol Chem* **274**, 896-902, doi:10.1074/jbc.274.2.896 (1999).

13 Smith, A. J. & Lippiat, J. D. Direct endosomal acidification by the outwardly rectifying CLC-5 Cl^-/H^+ exchanger. *J Physiol* **588**, 2033-2045, doi:10.1113/jphysiol.2010.188540 (2010).

14 Sato-Numata, K., Numata, T., Okada, T. & Okada, Y. Acid-sensitive outwardly rectifying (ASOR) anion channels in human epithelial cells are highly sensitive to temperature and independent of ClC-3. *Pflügers Arch* **465**, 1535-1543, doi:10.1007/s00424-013-1296-y (2013).

15 Li, X., Wang, T., Zhao, Z. & Weinman, S. A. The ClC-3 chloride channel promotes acidification of lysosomes in CHO-K1 and Huh-7 cells. *Am J Physiol Cell Physiol* **282**, C1483-1491, doi:10.1152/ajpcell.00504.2001 (2002).

16 Neagoe, I., Stauber, T., Fidzinski, P., Bergsdorf, E. Y. & Jentsch, T. J. The late endosomal ClC-6 mediates proton/chloride countertransport in heterologous plasma membrane expression. *J Biol Chem* **285**, 21689-21697, doi:10.1074/jbc.M110.125971 (2010).

17 Leisle, L., Ludwig, C. F., Wagner, F. A., Jentsch, T. J. & Stauber, T. ClC-7 is a slowly voltage-gated $2Cl^-/1H^+$-exchanger and requires Ostm1 for transport activity. *EMBO J* **30**, 2140-2152, doi:10.1038/emboj.2011.137 (2011).

18 Sato-Numata, K., Numata, T. & Okada, Y. Temperature sensitivity of acid-sensitive outwardly rectifying (ASOR) anion channels in cortical neurons is involved in hypothermic neuroprotection against acidotoxic necrosis. *Channels (Austin, Tex* **8**, 278-283, doi:10.4161/chan.27748 (2014).

19 Yang, J. *et al.* PAC, an evolutionarily conserved membrane protein, is a proton-activated chloride channel. *Science* **364**, 395-399, doi:10.1126/science.aav9739 (2019).
