# Supplementary material for: Proton-gated anion transport governs macropinosome shrinkage
Source: Nat Cell Biol. 2022 May 19;24(6):885–95. doi: 10.1038/s41556-022-00912-0 (PMC9203271; doi:10.1038/s41556-022-00912-0)
Supplement: Supplementary file 2 — Reporting Summary [file 41556_2022_912_MOESM2_ESM.pdf]

nature portfolio

Corresponding author(s): Prof. Thomas J. Jentsch

Last updated by author(s): Mar 28, 2022

Reporting Summary

Nature Portfolio wishes to improve the reproducibility of the work that we publish. This form provides structure for consistency and transparency in reporting. For further information on Nature Portfolio policies, see our Editorial Policies and the Editorial Policy Checklist.

Statistics

For all statistical analyses, confirm that the following items are present in the figure legend, table legend, main text, or Methods section.

n/a	Confirmed	
☐	☒	The exact sample size (n) for each experimental group/condition, given as a discrete number and unit of measurement
☐	☒	A statement on whether measurements were taken from distinct samples or whether the same sample was measured repeatedly
☐	☒	The statistical test(s) used AND whether they are one- or two-sided<br>*Only common tests should be described solely by name; describe more complex techniques in the Methods section.*
☒	☐	A description of all covariates tested
☐	☒	A description of any assumptions or corrections, such as tests of normality and adjustment for multiple comparisons
☐	☒	A full description of the statistical parameters including central tendency (e.g. means) or other basic estimates (e.g. regression coefficient) AND variation (e.g. standard deviation) or associated estimates of uncertainty (e.g. confidence intervals)
☐	☒	For null hypothesis testing, the test statistic (e.g. F, t, r) with confidence intervals, effect sizes, degrees of freedom and P value noted<br>*Give P values as exact values whenever suitable.*
☒	☐	For Bayesian analysis, information on the choice of priors and Markov chain Monte Carlo settings
☒	☐	For hierarchical and complex designs, identification of the appropriate level for tests and full reporting of outcomes
☐	☒	Estimates of effect sizes (e.g. Cohen's d, Pearson's r), indicating how they were calculated

Our web collection on statistics for biologists contains articles on many of the points above.

Software and code

Policy information about availability of computer code

Data collection	Live imaging of the macropinosomes was performed using Nikon spinning disc microscope (Yokogawa spinning disk 695 CSU-X1) operated with NIS software (version 5.02.01 Build 1270). Scratch assay was performed using Nikon Ti Eclipse microscope operated with NIS software (version 5.21.03 Build 1481). For immunocytochemistry pictures, the confocal microscope LSM880 (Zeiss, Zen Blue software 2.3) was used. Electrophysiological recordings were performed using an EPC-10 USB patch-clamp amplifier and PatchMaster software (HEKA Elektronik, version 2x90.3). For growth assay absorbance was measured with absorbance plate reader (ASYS Hitech).
Data analysis	All microscopy images were analysed using Fiji 2.0/2.1 versions. Custom data analysis code was written using Python 3.8 using OpenCV (version 4.2.0) and scikit-image (version 0.16.2) packages. Plotting and statistical analyses were performed with GraphPad Prism 7.03.

For manuscripts utilizing custom algorithms or software that are central to the research but not yet described in published literature, software must be made available to editors and reviewers. We strongly encourage code deposition in a community repository (e.g. GitHub). See the Nature Portfolio guidelines for submitting code & software for further information.

Data

Policy information about availability of data

All manuscripts must include a data availability statement. This statement should provide the following information, where applicable:

- Accession codes, unique identifiers, or web links for publicly available datasets
- A description of any restrictions on data availability
- For clinical datasets or third party data, please ensure that the statement adheres to our policy

Data is freely available from authors upon request. Code is freely avaliable on Github repository: mathematical model (https://github.com/mzeziulia/

MP_volume_modelling), macropinosome detection and analysis (https://github.com/mzeziulia/MP_detection_analysis), scratch assay analysis (https://github.com/mzeziulia/Scratch_assay)

Field-specific reporting

Please select the one below that is the best fit for your research. If you are not sure, read the appropriate sections before making your selection.

☒ Life sciences ☐ Behavioural & social sciences ☐ Ecological, evolutionary & environmental sciences

For a reference copy of the document with all sections, see nature.com/documents/nr-reporting-summary-flat.pdf

Life sciences study design

All studies must disclose on these points even when the disclosure is negative.

Sample size	Sample sizes were not predetermined with statistical means but were based on standard numbers in the field. Each experiment based on live cell microscopy was performed on at least 3 animals resulting in 10-800 vesicles analysed per condition. For immunocytochemistry, on average 15 cells per condition were analysed. Western Blot analyses were performed on 2-3 different animals. For growth assay 3 different knock-out cell lines generated with 2 different sgRNAs were used, experiments were repeated 3 times, each measurement in 4 technical replicates. For scratch assay 13 pairs of animals were used.
Data exclusions	No data were excluded.
Replication	All experiments were repeated independently on minimum 2 different animals with the same outcome, all obtained quantitative data is shown in figures. Key experiments based on live cell imaging were performed on 3 different TMEM206 knock out mouse lines. Experiments using cell lines were repeated independently on cells with different passage number with the same outcome. Growth assay was performed 3 times on 3 different knock-out cell lines generated with 2 different sgRNAs with the same outcome.
Randomization	No animal randomization was done because mice are assigned to their group based on genotype. Microscopic fields were randomly chosen when possible.
Blinding	Genotypes of animals for scratch assay and genotypes of MIA PaCa-2 cells for growth assay were blinded. No blinding was applied for macropinosome shrinkage assay since it was logistically impossible but microscopic fields were randomly chosen.<br>Key experiments based on live cell imaging were analyzed automatically by a custom code, minimizing potential analysis biais.

Reporting for specific materials, systems and methods

We require information from authors about some types of materials, experimental systems and methods used in many studies. Here, indicate whether each material, system or method listed is relevant to your study. If you are not sure if a list item applies to your research, read the appropriate section before selecting a response.

Materials & experimental systems

n/a	Involved in the study	
☐	☒	Antibodies
☐	☒	Eukaryotic cell lines
☒	☐	Palaeontology and archaeology
☐	☒	Animals and other organisms
☒	☐	Human research participants
☒	☐	Clinical data
☒	☐	Dual use research of concern

Methods

n/a	Involved in the study	
☒	☐	ChIP-seq
☒	☐	Flow cytometry
☒	☐	MRI-based neuroimaging

Antibodies

Antibodies used

The following antibodies were used :
anti-LAMP1 (rat, BD Pharmingen, 553792)
anti-EEA1 (mouse, Abcam, ab70521)
anti-EEA1 (sheep, R&D Systems, AF8047)
anti-Rab5A (mouse, Cell Signaling, 46449)
anti-Rab5 (mouse, BD Bioscience, BD610725)
anti-Rab7 (mouse, SCBT, sc-376362)
anti-GFP (chicken, Aves lab, GFP-1020)
anti-HA (rabbit, Cell Signaling, 3724)
anti-RFP (rabbit, Rockland, 600-401-379)
anti- α1 Na/K ATPase clone C464.6 (mouse, Millipore, 05–369)
anti-actin (mouse, Sigma, A2228)
anti-actin (rabbit, Sigma, A2066)

March 2021

anti-ClC-2 (rabbit, Jentsch lab)
anti-ClC-3 (rabbit, Jentsch lab)
anti-ClC-4 (rabbit, Jentsch lab)
anti-ClC-5 (rabbit or guinea pig, Jentsch lab)
anti-ClC-6 (rabbit, Jentsch lab)
anti-ClC-7 (rabbit, Jentsch lab)
anti-a3 subunit of H+ ATPase (guinea pig, Jentsch lab)
anti-TMEM206 against the Cterminus (rabbit, Jentsch lab)
anti-TMEM206 against the extracellular loop (rabbit, Jentsch lab)
anti-LRRC8A (rabbit, Jentsch lab)

Validation

All commercially available antibodies were validated by manufacturers and used in published papers (see manufacturers' websites for immunohistochemistry on western blot examples).
anti-LAMP1 antibody (rat, BD Pharmingen, 553792) was validated in https://www.citeab.com/antibodies/2410022-553792-bd-pharmingen-purified-rat-anti-mouse-cd107a
anti-EEA1 (mouse, Abcam, ab70521) was validated in https://www.abcam.com/eea1-antibody-1g11-early-endosome-marker-ab70521.html
anti-EEA1 (sheep, R&D Systems, AF8047) was validated in https://www.rndsystems.com/products/human-mouse-rat-eea1-antibody_af8047
anti-Rab5A (mouse, Cell Signaling, 46449) was validated in https://www.cellsignal.com/products/primary-antibodies/rab5a-e6n8s-mouse-mab/46449
anti-Rab5 (mouse, BD Bioscience, BD610725) was validated in https://www.bdbiosciences.com/en-de/products/reagents/microscopy-imaging-reagents/immunofluorescence-reagents/purified-mouse-anti-rab5.610725
anti-Rab7 (mouse, SCBT, sc-376362) was validated in https://www.scbt.com/p/rab-7-antibody-b-3?productCanUrl=rab-7-antibody-b-3&_requestid=10564832
anti-GFP (chicken, Aves lab, GFP-1020) was validated in https://www.aveslabs.com/products/anti-green-fluorescent-protein-antibody-gfp
anti-HA (rabbit, Cell Signaling, 3724) was validated in https://www.cellsignal.de/products/primary-antibodies/ha-tag-c29f4-rabbit-mab/3724
anti-RFP (rabbit, Rockland, 600-401-379) was validated in https://www.rockland.com/categories/primary-antibodies/rfp-antibody-pre-adsorbed-600-401-379/
anti- α1 Na/K ATPase clone C464.6 (mouse, Millipore, 05–369) was validated in https://www.merckmillipore.com/DE/de/product/Anti-Na-K-ATPase-1-Antibody-clone-C464.6,MM_NF-05-369?ReferrerURL=https%3A%2F%2Fwww.google.com%2F
anti-actin (mouse, Sigma, A2228) was validated in https://www.sigmaaldrich.com/DE/en/product/sigma/a2228
anti-actin (rabbit, Sigma, A2066) was validated in https://www.sigmaaldrich.com/DE/en/product/sigma/a2066

Newly generated custom-made TMEM206 antibodies were validated with Western Blot analysis and immunocytochemistry using HEK TMEM206 KO cells and tissues/cells from different Tmem206 knock-out mouse lines. All other custom-made antibodies (ClC-2, ClC-3, ClC-4, ClC-5, ClC-6, ClC-7, LRRC8A and a3 subunit of V-ATPase) were previously produced in our lab and were validated according to the field's highest standards by Western Blot analysis and immunohistochemistry on tissues from respective knock-out mouse lines (see methods and reference sections in the manuscript).

Eukaryotic cell lines

Policy information about cell lines

Cell line source(s)

Wild-type HEK293, HeLa, HT-1080 and MIA PaCa-2 are from the Deutsche Sammlung von Mikroorganismen und Zellkulturen, Germany.

Authentication

Parental cells of all above mentioned cells lines were purchased from the Deutsche Sammlung von Mikroorganismen und Zellkulturen, Germany.

Mycoplasma contamination

In all experiments cells were mycoplasma negative.

Commonly misidentified lines
(See ICLAC register)

No commonly misidentified lines were used.

Animals and other organisms

Policy information about studies involving animals; ARRIVE guidelines recommended for reporting animal research

Laboratory animals

C57BL/6 Mice (Mus musculus) aged of 8-22 weeks (8-16 weeks for BMDMs preparation) and from both sexes were used in this study.
The following lines were used : B6;Lrrc8atm2c(EUCOMM)Hmgu-em2(HA)Tjj
B6;Lrrc8dem1(tdtomato; loxP)Tjj
B6;129/Sv-Clcn2tm1Tjj
B6;129/Sv-Clcn3tm1Tjj
B6;129/Sv-Clcn4tm1Tjj
B6;129/Sv-Clcn5tm1Tjj
B6;129/Sv-Clcn7tm3.1Tjj x Cx3cr1CreER x ROSA26floxSTOP-YFP
B6;Lrrc8atm2a(EUCOMM)Hmgu x Cx3cr1CreER
and the 3 newly generated TMEM206 knock-out lines (as described in the methods section).

Wild animals

No wild animals were used.

Field-collected samples	No samples were collected from the field.
Ethics oversight	All animal experiments and the generation of new mouse lines were approved by Berlin authorities (LAGeSo, G0005/19 licence).

Note that full information on the approval of the study protocol must also be provided in the manuscript.
