# Supplementary figures and images for: Proton-gated anion transport governs macropinosome shrinkage
Source: Nat Cell Biol. 2022 May 19;24(6):885–95. doi: 10.1038/s41556-022-00912-0 (PMC9203271; doi:10.1038/s41556-022-00912-0)

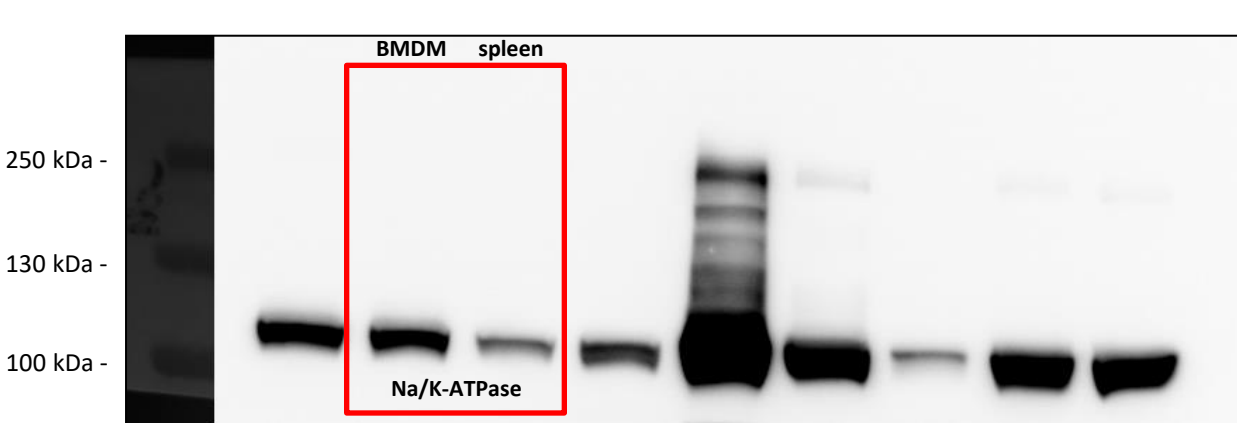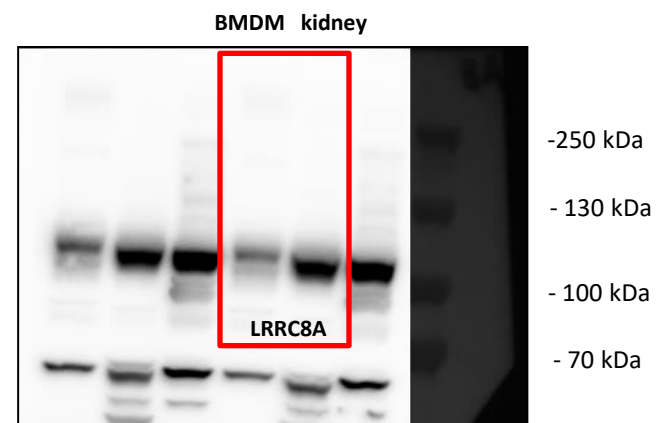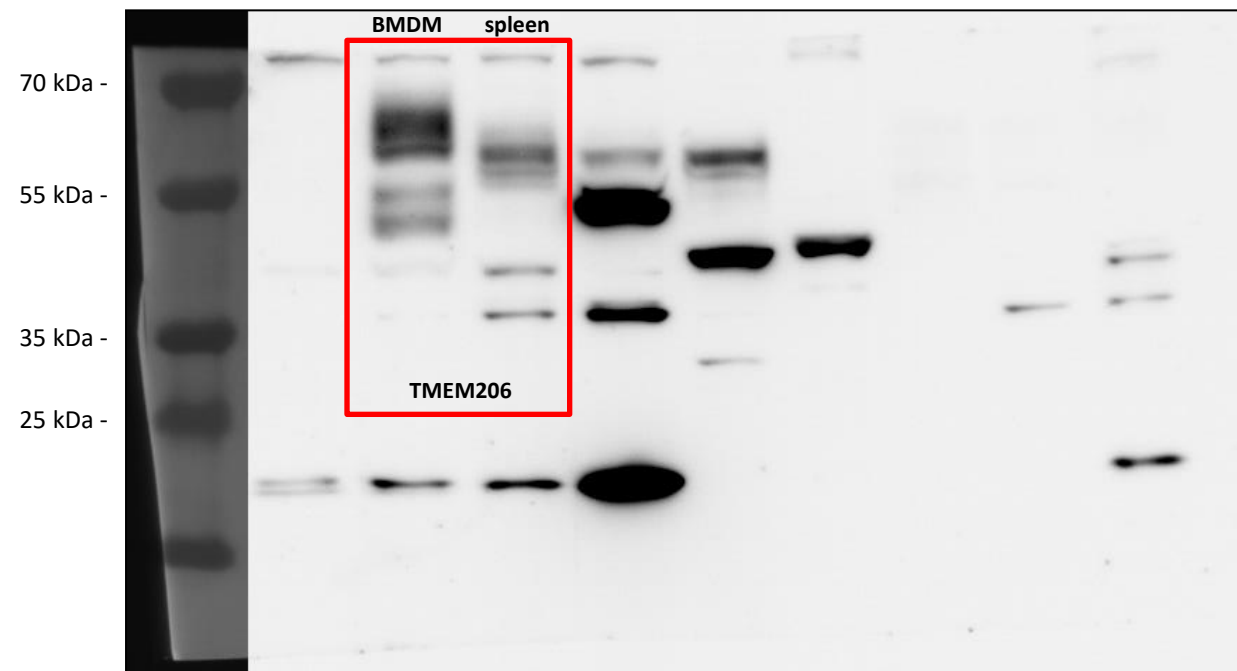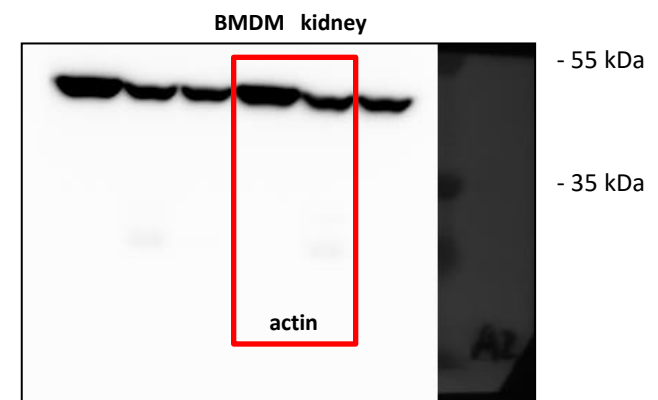

**Figure 1g**

**Figure 1f**

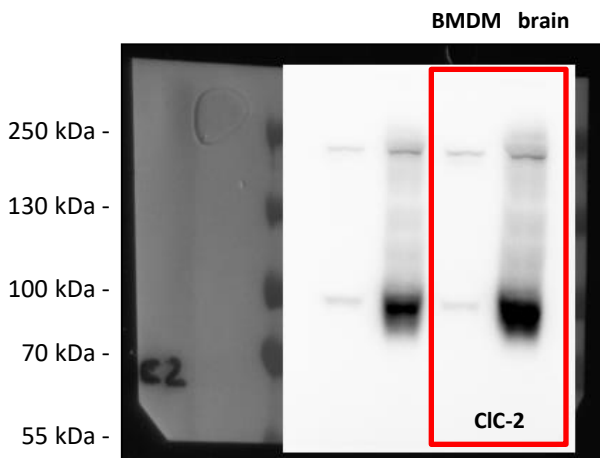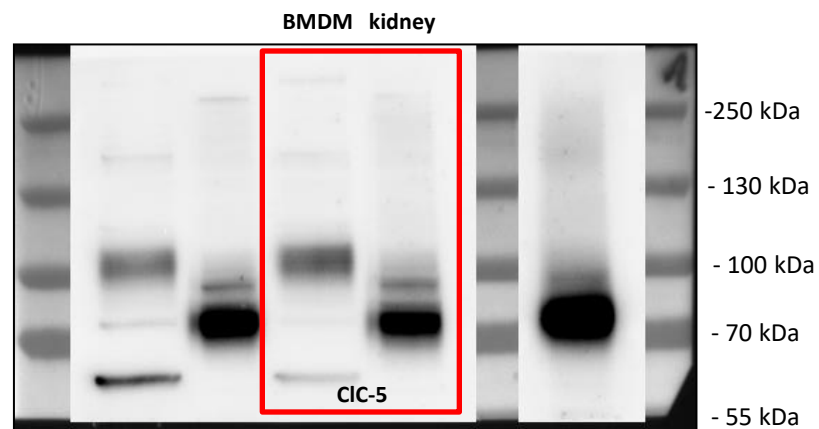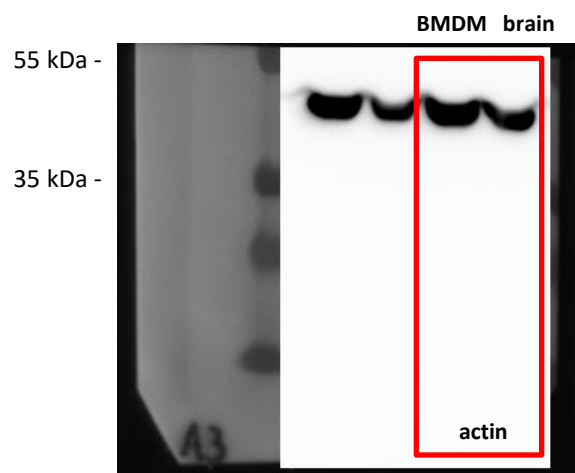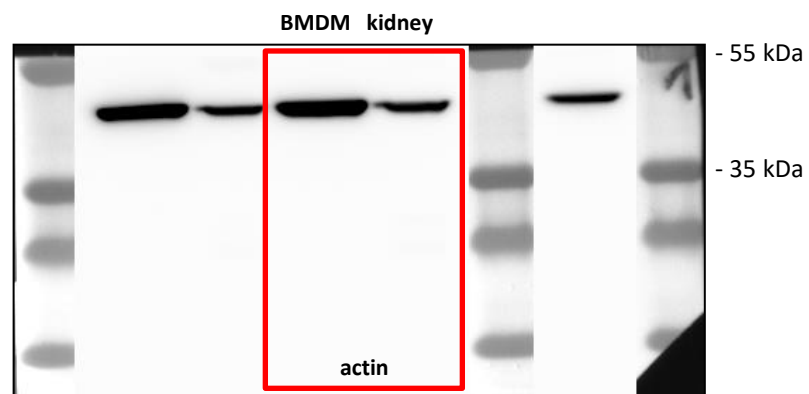

**Figure 1h**

**Figure 1k**

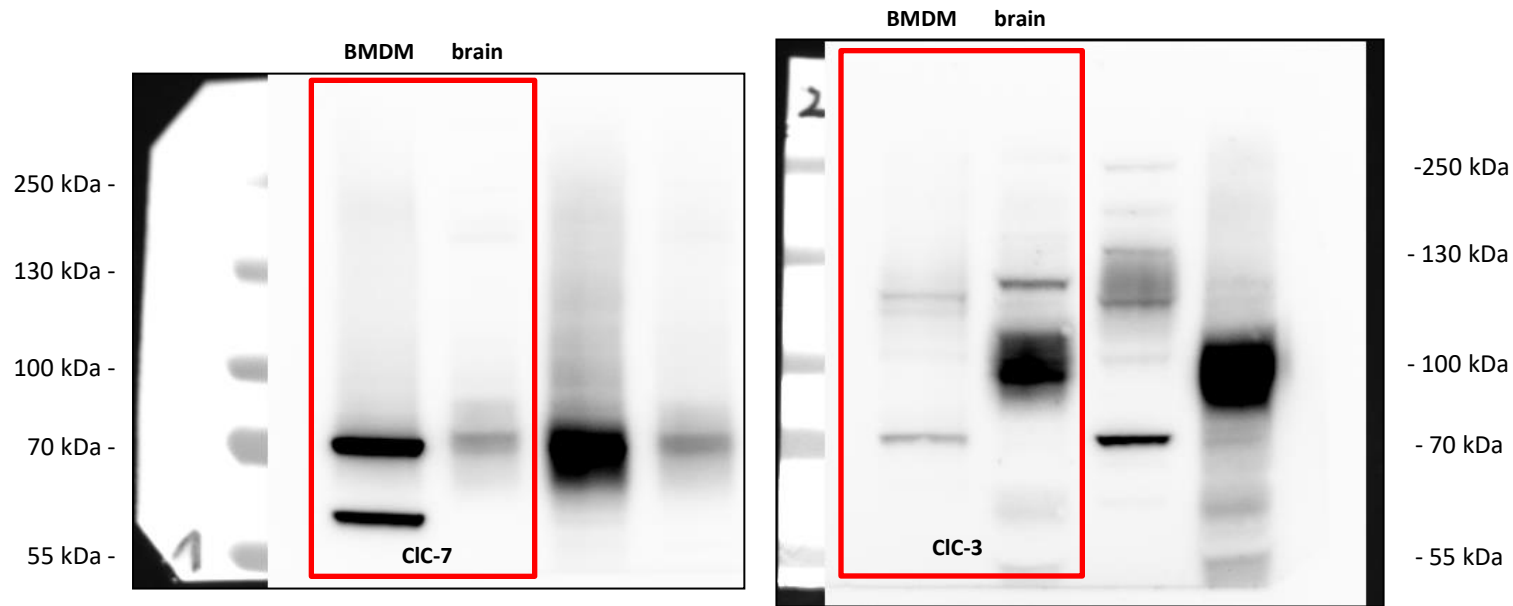

Figure 1m

Figure 1i

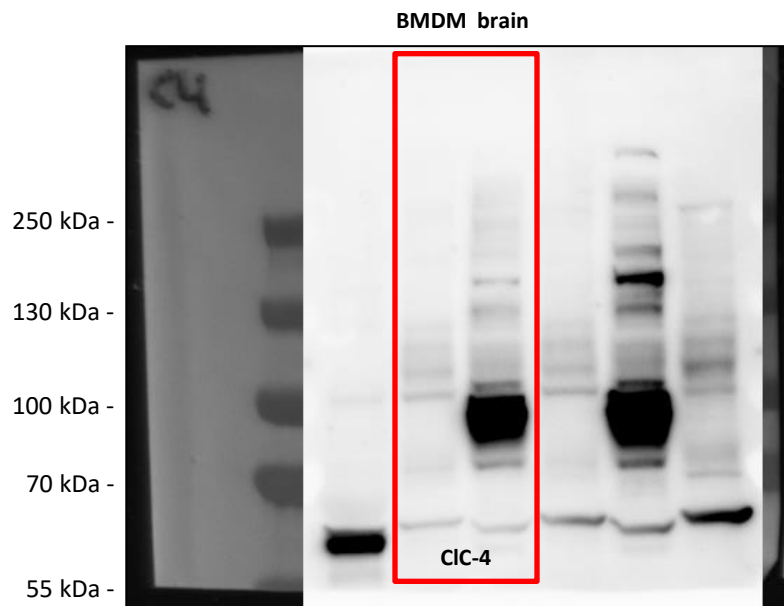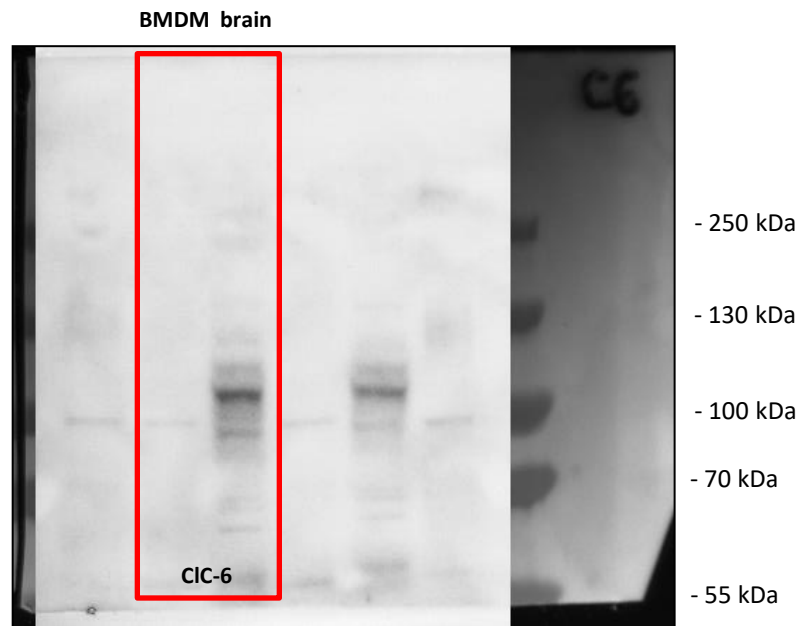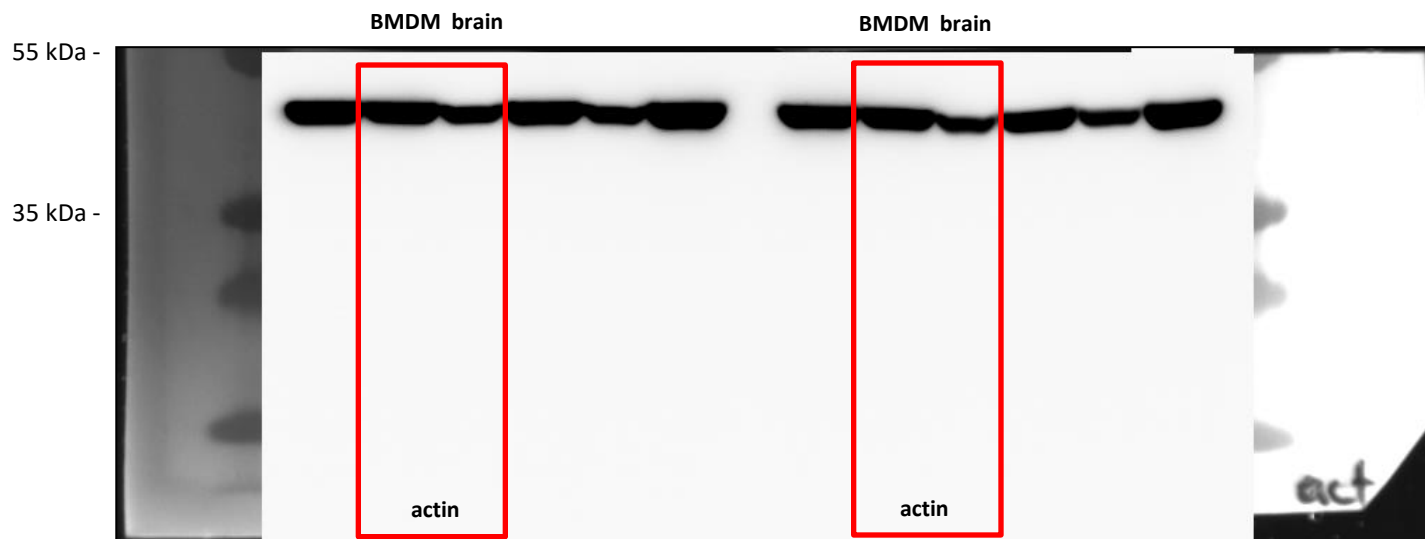

**Figure 1j**

**Figure 1l**

Supplement: Source Data Fig. 1 — Unprocessed western blots. [file 41556_2022_912_MOESM8_ESM.pdf]

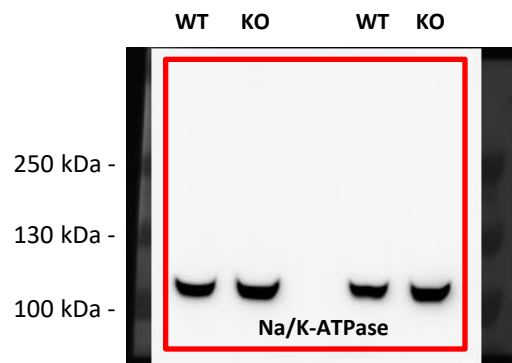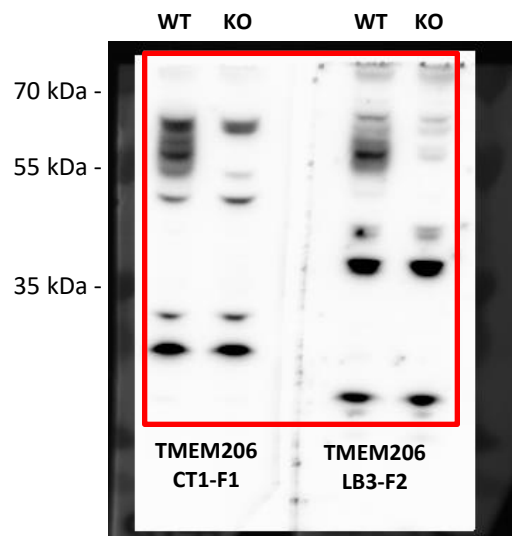

Extended Data Figure 1a+b

Supplement: Source Data Extended Data Fig. 1 — Unprocessed western blots. [file 41556_2022_912_MOESM15_ESM.pdf]

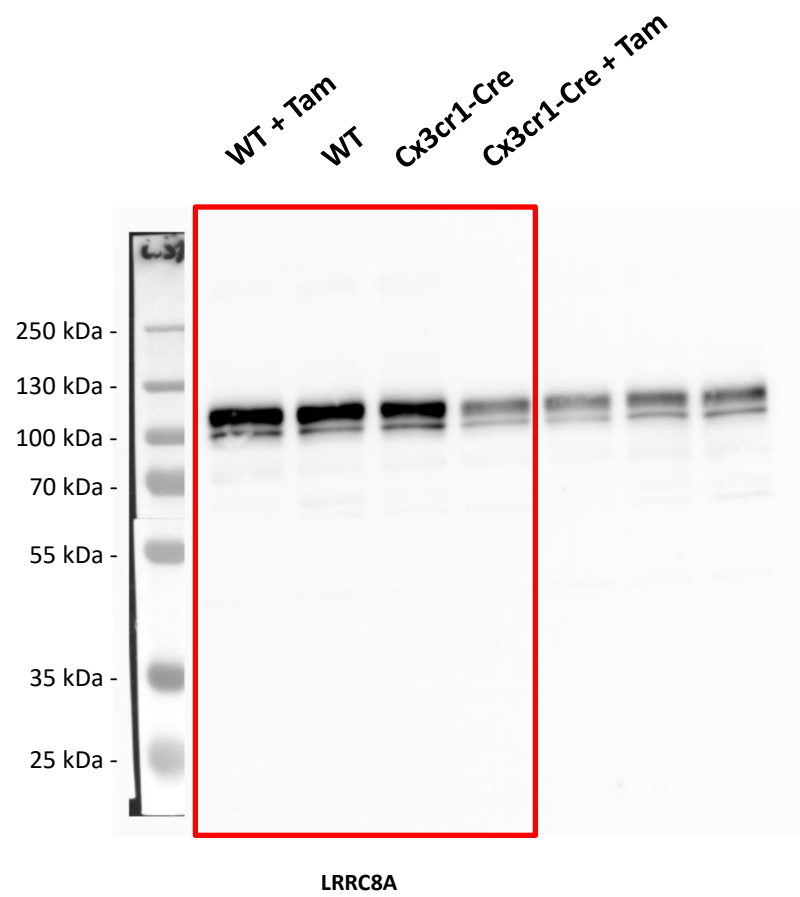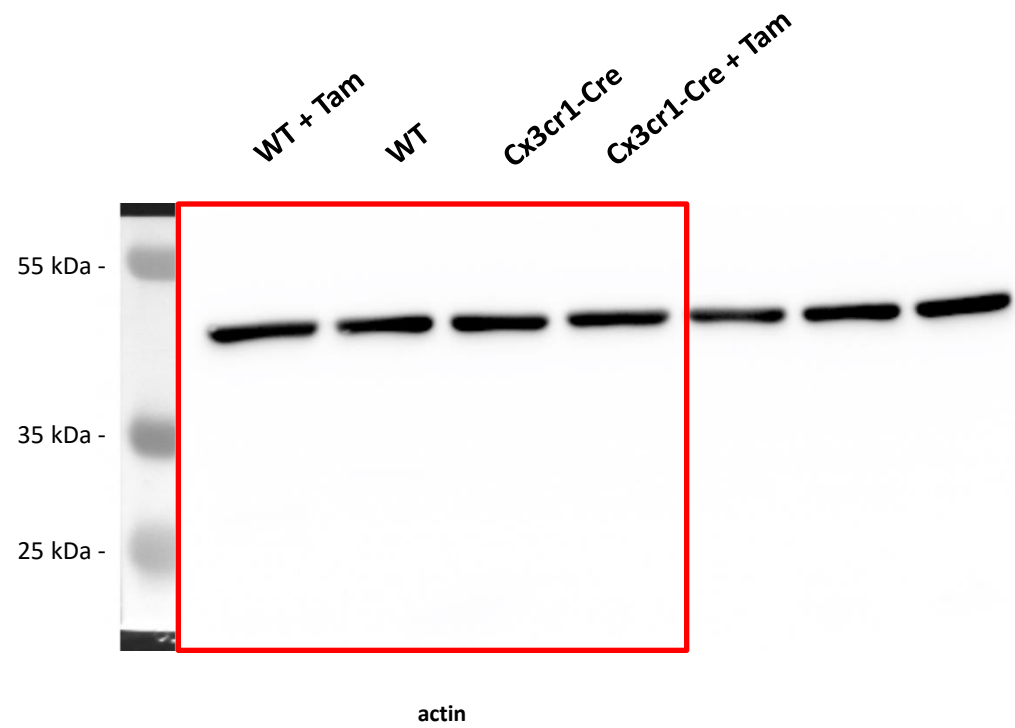

Supplement: Source Data Extended Data Fig. 5 — Unprocessed western blots. [file 41556_2022_912_MOESM20_ESM.pdf]
