# Supplementary material for: Proton-gated anion transport governs macropinosome shrinkage
Source: Nat Cell Biol. 2022 May 19;24(6):885–95. doi: 10.1038/s41556-022-00912-0 (PMC9203271; doi:10.1038/s41556-022-00912-0)
Supplement: Source Data Extended Data Fig. 4 — Unprocessed western blots. [file 41556_2022_912_MOESM18_ESM.pdf]

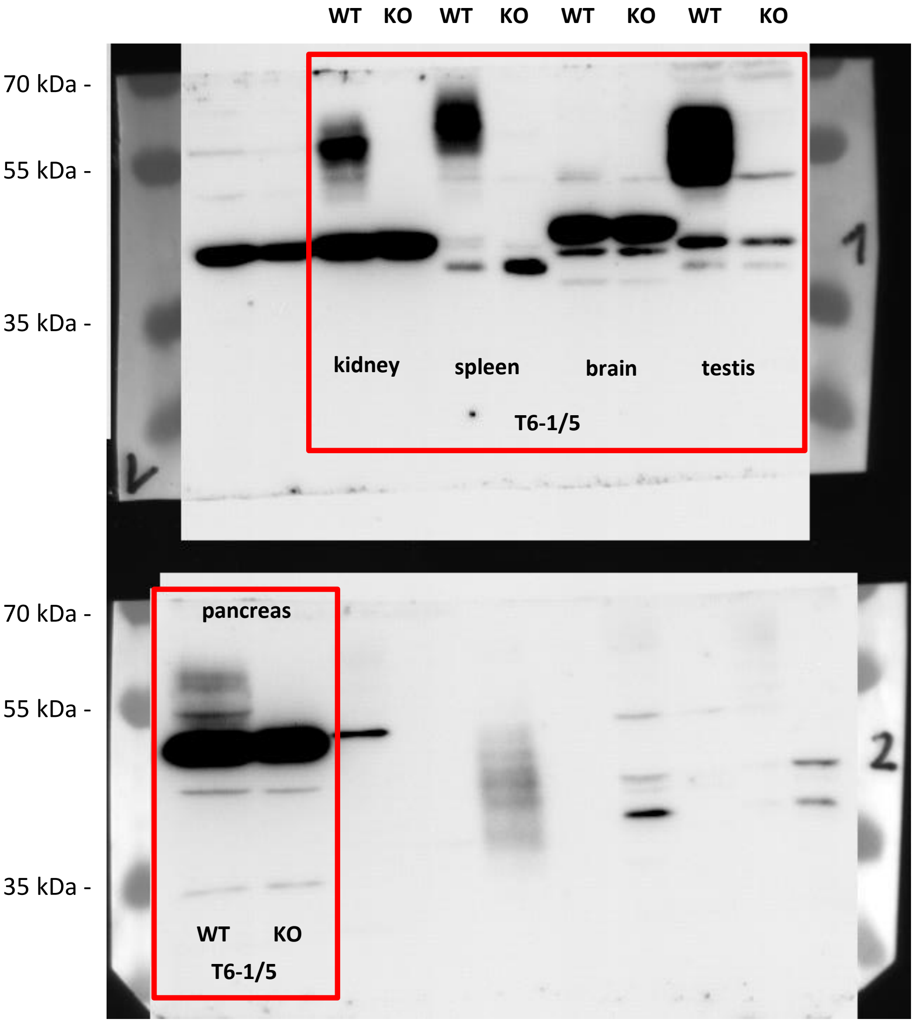

Extended Data Figure 4d

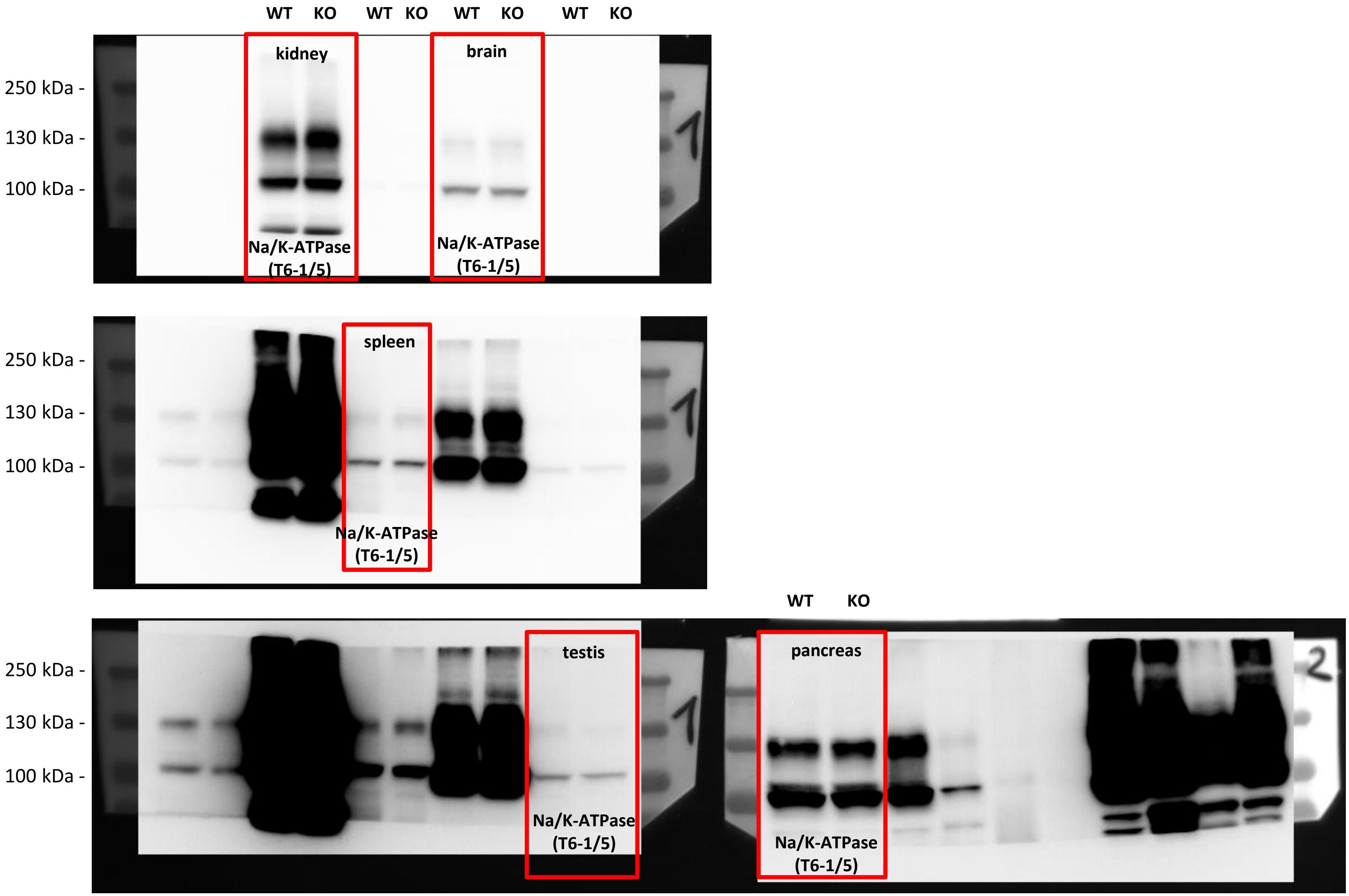

Extended Data Figure 4d

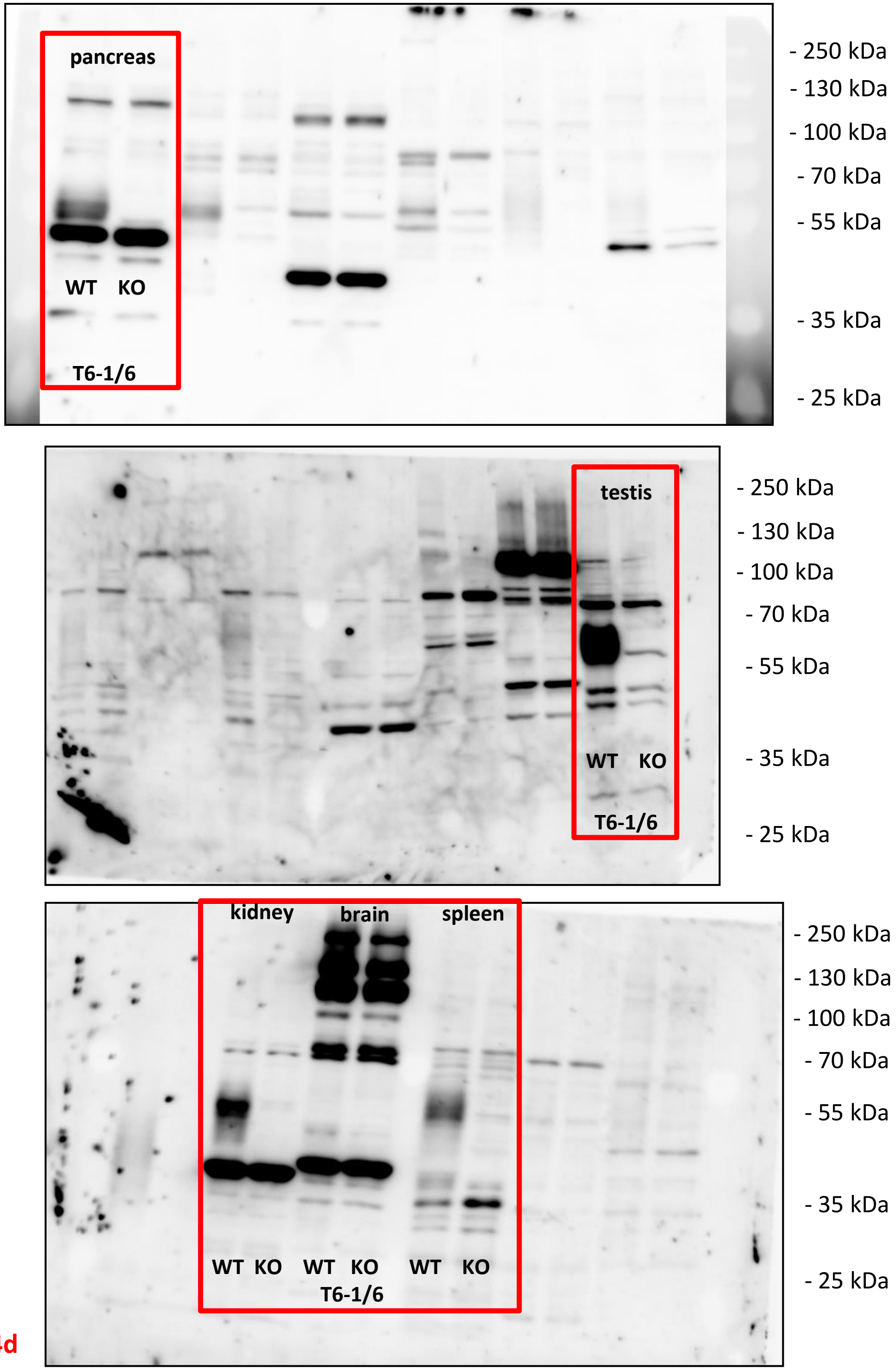

Extended Data Figure 4d

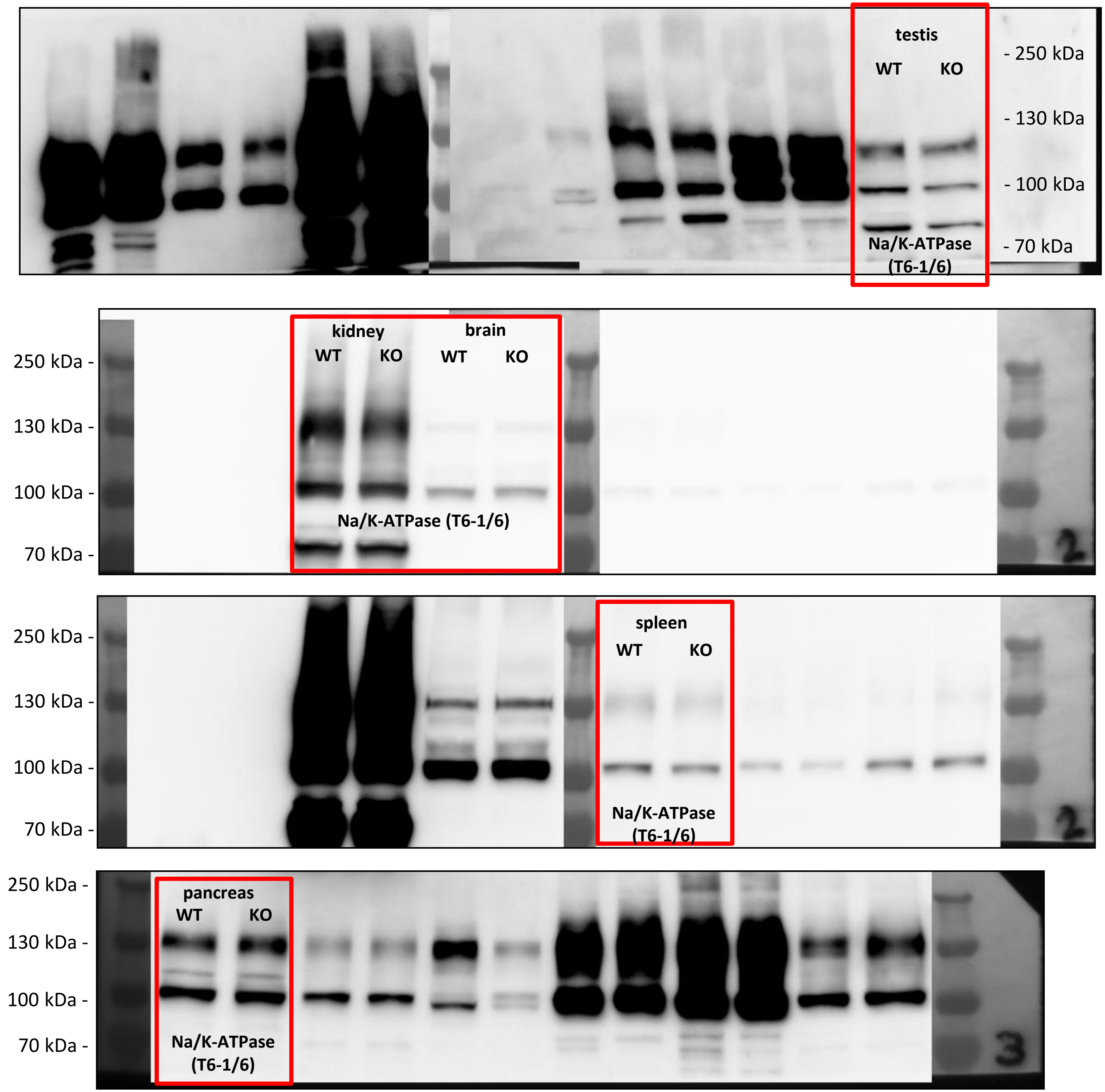

Extended Data Figure 4d

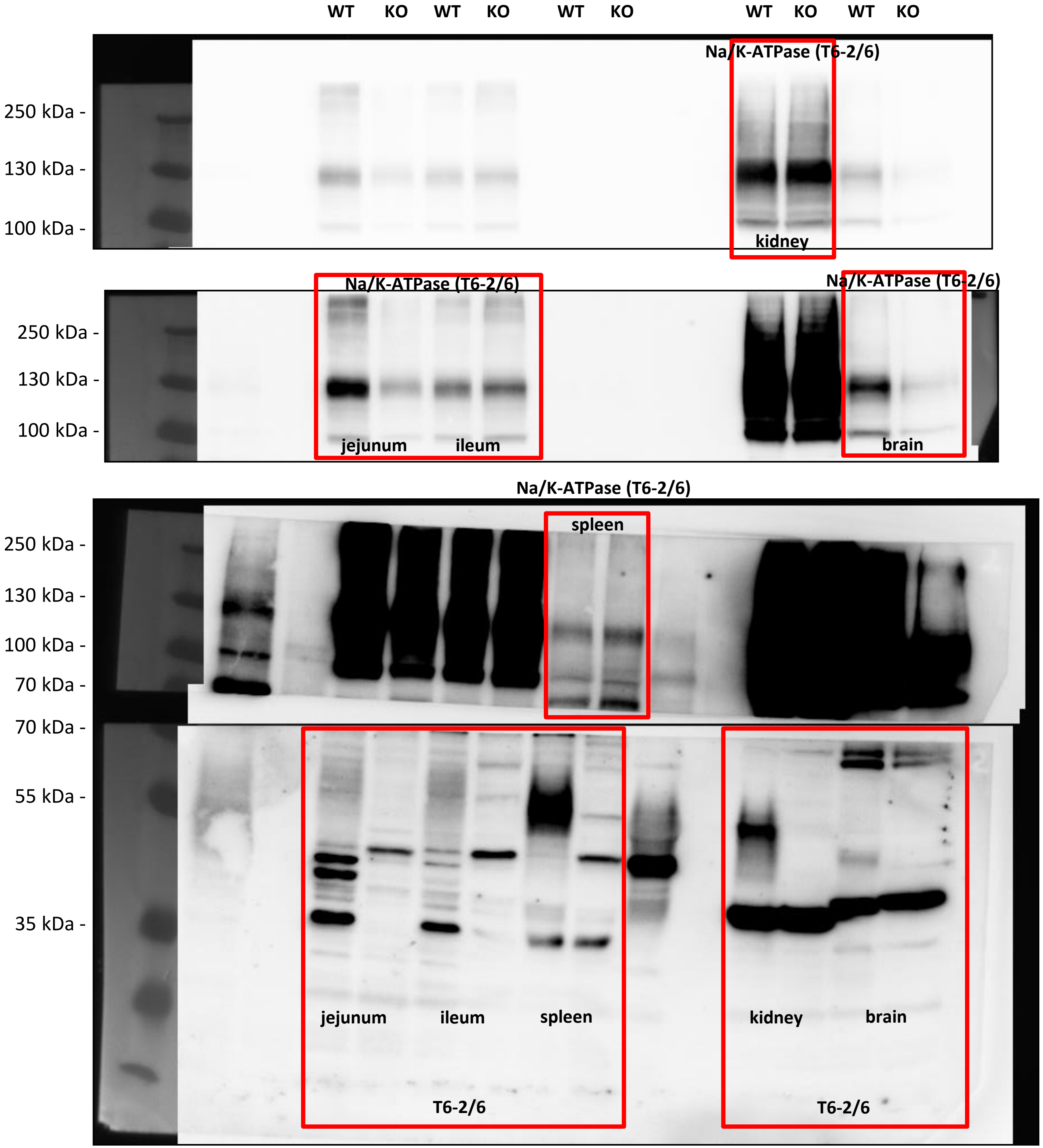

Extended Data Figure 4d

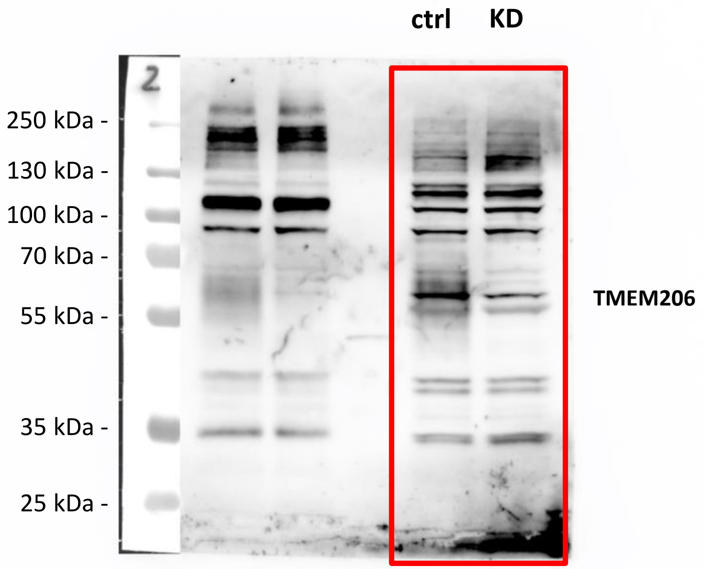

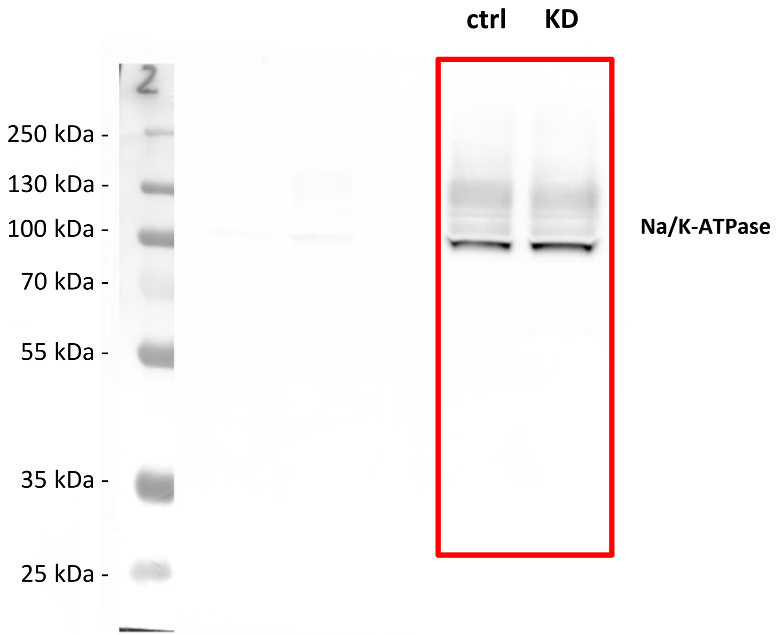

Extended Data Figure 4e
